# Ovarian Carcinoma Subtypes Are Different Diseases: Implications for Biomarker Studies

**DOI:** 10.1371/journal.pmed.0050232

**Published:** 2008-12-02

**Authors:** Martin Köbel, Steve E Kalloger, Niki Boyd, Steven McKinney, Erika Mehl, Chana Palmer, Samuel Leung, Nathan J Bowen, Diana N Ionescu, Ashish Rajput, Leah M Prentice, Dianne Miller, Jennifer Santos, Kenneth Swenerton, C. Blake Gilks, David Huntsman

**Affiliations:** 1 Genetic Pathology Evaluation Centre of the Prostate Research Centre, Department of Pathology, Vancouver General Hospital and British Columbia Cancer Agency, Vancouver, British Columbia, Canada; 2 Institute of Pathology, Charité Hospital, Berlin, Germany; 3 Canary Foundation, San Jose, California, United States of America; 4 School of Biology, Georgia Institute of Technology, and Ovarian Cancer Institute, Atlanta, Georgia, United States of America; 5 Department of Gynecology, Vancouver General Hospital and British Columbia Cancer Agency, Vancouver, British Columbia, Canada; 6 Cheryl Brown Ovarian Cancer Outcomes Unit, British Columbia Cancer Agency, Vancouver, British Columbia, Canada; Centre for Research in Women's Health, Canada

## Abstract

**Background:**

Although it has long been appreciated that ovarian carcinoma subtypes (serous, clear cell, endometrioid, and mucinous) are associated with different natural histories, most ovarian carcinoma biomarker studies and current treatment protocols for women with this disease are not subtype specific. With the emergence of high-throughput molecular techniques, distinct pathogenetic pathways have been identified in these subtypes. We examined variation in biomarker expression rates between subtypes, and how this influences correlations between biomarker expression and stage at diagnosis or prognosis.

**Methods and Findings:**

In this retrospective study we assessed the protein expression of 21 candidate tissue-based biomarkers (CA125, CRABP-II, EpCam, ER, F-Spondin, HE4, IGF2, K-Cadherin, Ki-67, KISS1, Matriptase, Mesothelin, MIF, MMP7, p21, p53, PAX8, PR, SLPI, TROP2, WT1) in a population-based cohort of 500 ovarian carcinomas that was collected over the period from 1984 to 2000. The expression of 20 of the 21 biomarkers differs significantly between subtypes, but does not vary across stage within each subtype. Survival analyses show that nine of the 21 biomarkers are prognostic indicators in the entire cohort but when analyzed by subtype only three remain prognostic indicators in the high-grade serous and none in the clear cell subtype. For example, tumor proliferation, as assessed by Ki-67 staining, varies markedly between different subtypes and is an unfavourable prognostic marker in the entire cohort (risk ratio [RR] 1.7, 95% confidence interval [CI] 1.2%–2.4%) but is not of prognostic significance within any subtype. Prognostic associations can even show an inverse correlation within the entire cohort, when compared to a specific subtype. For example, WT1 is more frequently expressed in high-grade serous carcinomas, an aggressive subtype, and is an unfavourable prognostic marker within the entire cohort of ovarian carcinomas (RR 1.7, 95% CI 1.2%–2.3%), but is a favourable prognostic marker within the high-grade serous subtype (RR 0.5, 95% CI 0.3%–0.8%).

**Conclusions:**

The association of biomarker expression with survival varies substantially between subtypes, and can easily be overlooked in whole cohort analyses. To avoid this effect, each subtype within a cohort should be analyzed discretely. Ovarian carcinoma subtypes are different diseases, and these differences should be reflected in clinical research study design and ultimately in the management of ovarian carcinoma.

## Abstract 2

David Huntsman and colleagues describe the associations between biomarker expression patterns and survival in different ovarian cancer subtypes. They suggest that the management of ovarian cancer should reflect differences between these subtypes.

## Editors' Summary

**Background.:**

Every year, about 200,000 women develop ovarian cancer and more than 100,000 die from the disease. Ovarian epithelial cancer (carcinoma) occurs when epithelial cells from the ovary or fallopian tube acquire mutations or equivalent changes that allow them to grow uncontrollably within one of the ovaries (two small organs in the pelvis that produce eggs) and acquire the potential to spread around the body (metastasize). While the cancer is confined to the ovaries, cancer specialists call this stage I disease; 70%–80% of women diagnosed with stage I ovarian cancer survive for at least 5 y. However, only a fifth of ovarian cancers are diagnosed at this stage; in the majority of patients the cancer has spread into the pelvis (stage II disease), into the peritoneal cavity (the space around the gut, stomach, and liver; stage III disease), or metastasized to distant organs such as brain (stage IV disease). This peritoneal spread might be associated with often only vague abdominal pain and mild digestive disturbances. Patients with advanced-stage ovarian carcinoma are treated with a combination of surgery and chemotherapy but, despite recent advances in treatment, only 15% of women diagnosed with stage IV disease survive for 5 y.

**Why Was This Study Done?:**

Although it is usually regarded as a single disease, there are actually several distinct subtypes of ovarian carcinoma. These are classified according to their microscopic appearance as high-grade serous, low-grade serous, clear cell, endometrioid, and mucinous ovarian carcinomas. These subtypes develop differently and respond differently to chemotherapy. Yet scientists studying ovarian carcinoma usually regard this cancer as a single entity, and current treatment protocols for the disease are not subtype specific. Might better progress be made toward understanding ovarian carcinoma and toward improving its treatment if each subtype were treated as a separate disease? Why are some tumors confined to the ovary, whereas the majority spread beyond the ovary at time of diagnosis? In this study, the researchers address these questions by asking whether correlations between the expression of “biomarkers” (molecules made by cancer cells that can be used to detect tumors and to monitor treatment effectiveness) and the stage at diagnosis or length of survival can be explained by differential biomarker expression between different subtypes of ovarian carcinoma. They also address the question of whether early stage and late stage ovarian carcinomas are fundamentally different.

**What Did the Researchers Do and Find?:**

The researchers measured the expression of 21 candidate protein biomarkers in 500 ovarian carcinoma samples collected in British Columbia, Canada, between 1984 and 2000. For 20 of the biomarkers, the fraction of tumors expressing the biomarker varied significantly between ovarian carcinoma subtypes. Considering all the tumors together, ten biomarkers had different expression levels in early and late stage tumors. However, when each subtype was considered separately, the expression of none of the biomarkers varied with stage. When the researchers asked whether the expression of any of the biomarkers correlated with survival times, they found that nine biomarkers were unfavorable indicators of outcome when all the tumors were considered together. That is, women whose tumors expressed any of these biomarkers had a higher risk of dying from ovarian cancer than women whose tumors did not express these biomarkers. However, only three biomarkers were unfavorable indicators for high-grade serous carcinomas considered alone and the expression of a biomarker called WT1 in this subtype of ovarian carcinoma is associated with a lower risk of dying. Similarly, expression of the biomarker Ki-67 was an unfavorable prognostic indicator when all the tumors were considered, but was not a prognostic indicator for any individual subtype.

**What Do These Findings Mean?:**

These and other findings indicate that biomarker expression is more strongly associated with ovarian carcinoma subtype than with stage. In other words, biomarker expression is constant from early to late stage, but only within a given subtype. Second, the association of biomarker expression with survival varies between subtypes, hence lumping all subtypes together can yield misleading results. Although these findings need confirming in more tumor samples, they support the view that ovarian carcinoma subtypes are different diseases. In practical terms, therefore, these findings suggest that better ways to detect and treat ovarian cancer are more likely to be found if future biomarker studies and clinical research studies investigate each subtype of ovarian carcinoma separately rather than grouping them all together.

**Additional Information.:**

Please access these Web sites via the online version of this summary at http://dx.doi.org/10.1371/journal.pmed.0050232.

- The US National Cancer Institute provides a brief description of what cancer is and how it develops and information on all aspects of ovarian cancer for patients and professionals. It also provides a fact sheet on tumor markers (in English and Spanish)
- The UK charity Cancerbackup provides general information about cancer and more specific information about ovarian cancer, including tumor staging

## Introduction

Ovarian carcinoma is a heterogeneous disease. On the basis of histopathological examination, pathologists classify ovarian carcinoma into serous, clear cell, endometrioid, and mucinous subtypes. Each of theses subtypes is associated with different genetic risk factors and molecular events during oncogenesis [1,2], and characterized by distinct mRNA expression profiles [3,4]. These subtypes differ dramatically in frequency, when early stage carcinomas (where the majority are nonserous carcinomas [5]) and advanced stage carcinomas (which are predominantly of serous subtype [6]) are compared.

Oncologists have noted that subtypes respond differently to chemotherapy. The dismal response rate of clear cell carcinomas (15%) contrasts sharply with that of high-grade serous (80%), resulting in a lower 5-y survival for clear cell compared with high-grade serous carcinoma in patients with advanced stage tumors (20% versus 30%) [7,8]. Therefore, the National Cancer Institute (NCI) State of Science meeting recently singled out clear cell carcinoma as a candidate for clinical trials to identify more active therapy than what is currently available [9]. Although these data suggest substantial differences between subtypes, ovarian carcinoma is typically approached as a monolithic entity by researchers and clinicians. This practice impedes progress in understanding the biology or improving the management of the less common ovarian carcinoma subtypes.

We hypothesized that correlations between biomarker expression and stage at diagnosis or prognosis would reflect subtype variation in biomarker expression. To test this hypothesis we correlated protein expression rates of a panel of 21 candidate biomarkers with stage at diagnosis and disease-specific survival (DSS) in a large cohort of ovarian carcinomas and also analyzed these associations within ovarian carcinoma subtypes.

## Methods

### Study Population

The Cheryl Brown Ovarian Cancer Outcomes Unit is an ovarian cancer registry serving a population of approximately four million people in British Columbia. For the period 1984–2000, 2,555 patients with ovarian carcinoma were recorded in the registry. From these 834 patients were selected based on the criterion being free of macroscopic apparent residual disease after primary surgery and all histological slides underwent gynecopathological review. Subtypes were assigned according to refined World Health Organization (WHO) criteria [10] as recently described [5]. A further 91 patients diagnosed in stage 1a or 1b, grade 1 were excluded from the study because of excellent prognosis; only 3% of women in this group died of disease during the follow-up period. From the remaining patients 541 tissue blocks were available and used for tissue microarray (TMA) construction. A representative area of each tumor was selected and duplicate 0.6-mm tissue cores were punched to construct a TMA (Beecher Instruments). Review after TMA construction revealed that 23 cases were not adequately sampled. Of these 23 cases, 20 mixed carcinomas (>10% of tumor showing a second histological cell type) were excluded because their highest grade component was not sampled on the TMA; 18 cases were either of rare histological types (including seven undifferentiated, six transitional, and one squamous carcinoma) or could not be specified (five cases). This approach resulted in a study population of exactly 500 cases belonging to one of the four major cell types (serous, endometrioid, clear cell, and mucinous) (Table 1). The serous subtype was further subdivided into low- and high-grade [11]. Two cases of endometrioid carcinomas containing minor mucinous or low-grade serous components (>10%) are included in the study.

**Table 1:**
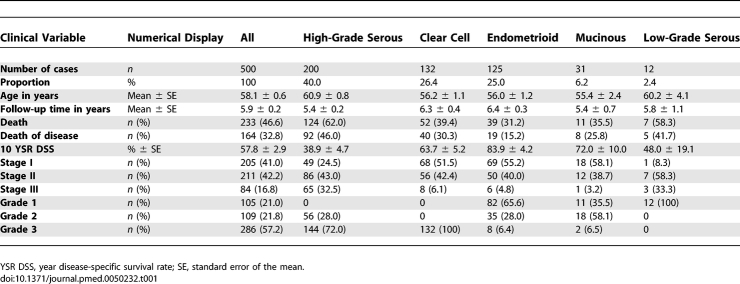
Study Population

### Adjuvant Therapy and Follow-up

All patients received standardized treatment according to the provincial treatment guidelines of the British Columbia Cancer Agency (BCCA) [12,13]; however, 3% of patients refused the advised adjuvant chemotherapy and were excluded from survival analysis. For 3% adjuvant therapy was not advised, hence 94% received platinum-based chemotherapy (with or without abdomino-pelvic radiotherapy) adjuvant treatments. Outcomes were tracked via the Cheryl Brown Ovarian Cancer Outcomes Unit at the BCCA and were available for all patients. Follow-up information was obtained through the electronic patient record of the BCCA or the patient's paper chart. Examples of documentation used to ascertain vital status include BCCA progress notes, death certificates, and correspondence indicating status from other care providers. Ovarian carcinoma specific death was defined where ovarian cancer was the primary or underlying cause of death. Death from concurrent disease (i.e., second malignancy) was coded as “died of other cause.” Death resulting from toxicities relating to treatments for ovarian carcinoma was coded as “died of toxicities.” Abstracted data were reviewed by an experienced medical oncologist (K.S.). Median follow-up time was 5.1 y. Approval for the study was obtained from the Research Ethics Board of the University of British Columbia.

### Marker Selection and Immunohistochemistry

The goal of our marker selection was to use proteins that are consistently expressed in ovarian carcinomas and have been reported as prognosticators (p53, p21, Ki-67, PR, WT1) [14–19] or being developed as early detection markers in ovarian carcinomas [20]. This approach biased our results towards selection of markers mostly derived from and expressed in high-grade serous subtype. Serial 4-μm sections were cut for immunohistochemical (IHC) analysis and run through an automated protocol including heat antigen retrieval (Ventana System). The antibodies and suppliers are listed in Table 2. Specificity was determined by using appropriate positive controls, with omission of primary antibody as a negative control.

**Table 2:**
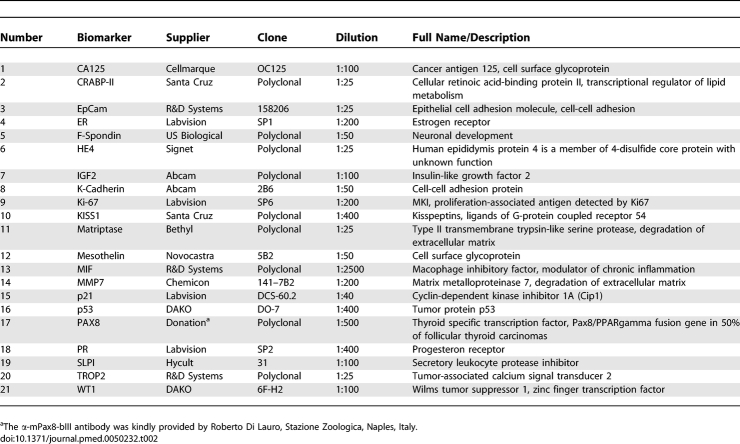
Antibodies

### Evaluation of Immunohistochemistry

One or more pathologists (MK, DNI, or AR) scored these biomarkers after scanning with a BLISS scanner (Bacus Laboratories/Olympus America). Except KISS1 [21] and p53 [22] where recently published cut-off points were used, all markers were dichotomized into negative and positive cases (cut-off values for positive versus negative for all markers except Ki-67 are shown in Table S1). Ki-67 was assessed as a continuous variable as a percentage of positive tumor cells using automated image analysis software [23]. Prior to analysis a pathologist (MK) manually selected regions of interest so as to avoid noncancerous cellular areas. The median was used to dichotomize into low- and high-expressing groups for Ki-67.

### Statistical Analysis

Contingency analysis and Pearson's Chi^2^ statistic were used to test the change in the distribution of biomarker expression across stage and subtypes. The Kruskal-Wallis test was used to determine if Ki-67 was differentially expressed across stage and subtypes. Univariable DSS was illustrated by the generation of Kaplan-Meier curves and subgroup differences tested with a univariable Cox model. Multivariable DSS was tested using the Cox proportional hazards model. The Cox proportional hazards model was used to determine risk ratios (RRs) and *p*-values for all univariable and multivariable DSS analyses. Additionally, to assess significance in the presence of some small subgroups, permutation tests were performed and permutation *p*-values reported. Under the null hypothesis of no association of biomarker status with survival (for survival analyses) or stage/histology (for contingency table analyses), the biomarker outcomes are exchangeable across cases. For the survival analyses, permutations of biomarker outcomes were performed within stage/subtype subgroups, to preserve the observed distribution of biomarker frequencies within subgroups. Permutation was performed by exchanging each case's entire biomarker panel at random without replacement among cases, to preserve correlation structure within case. A total of 10,000 permutation replications were performed. *p*-Values were obtained by finding the number of permutation sample estimates (Cox model parameter estimate for survival analyses, Pearson Chi^2^ statistic for contingency table analyses) as extreme or more extreme than the observed value. *p* < 0.05 was considered statistically significant. Hence, any prognostic correlations for a single biomarker have to be interpreted with caution. Statistical analyses were performed using SPSS software (version 15.0; SPSS) and R (version 2.5.1; R Foundation for Statistical Computing).

## Results

### Biomarker Expression Profile Reflects Subtype

This cohort of 500 ovarian carcinomas was mainly selected based on the criterion of not having apparent residual tumor after primary surgery. Since successful surgery is typically achieved in lower stage, this case selection strategy can be anticipated to include more cases of tumors of histological subtypes that are commonly diagnosed at low stage, such as clear cell carcinoma (26.4%), endometrioid (25.0%), and mucinous (6.2%) carcinomas, although serous carcinomas were still the most common subtype (40.0% high-grade and 2.4% low-grade) in this cohort (Table 1).

**Figure 1:**
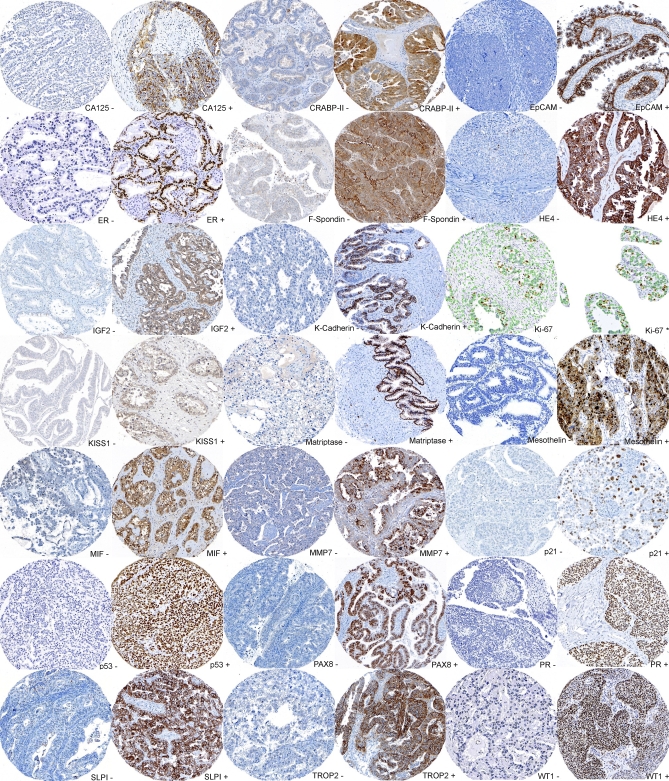
Representative Immunostains Paired positive and negative examples for each biomarker.

**Figure 2:**
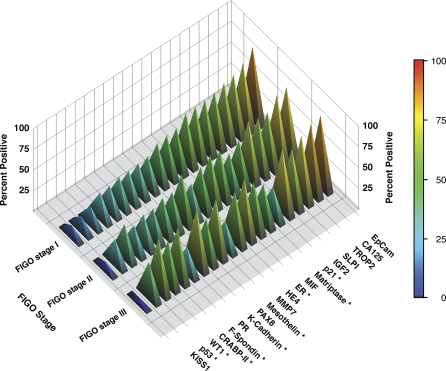
Biomarker Expression Rates in the Entire Cohort by Stage *Significant differences between categories (Fisher's exact test).

**Figure 3:**
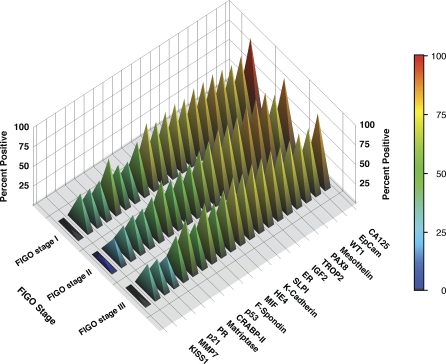
Biomarker Expression Rates in High-Grade Serous Subtype by Stage

**Figure 4:**
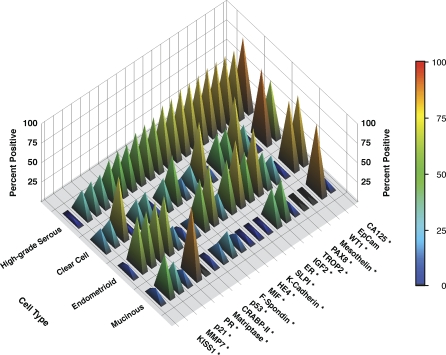
Biomarker Expression Rates in the Entire Cohort by Subtype *Significant differences between categories (Fisher's exact test). Note that the order in which biomarkers are presented is based on percentage of positivity and that therefore the order is different in Figures 2–4.

Interpretable results of immunostains for the 21 candidate biomarkers (Figure 1) ranged from 363 to 493 (median 488, Table S2). The larger numbers of missing data for three biomarkers were caused by exhaustion of tumor material in the core. All immunostains with annotated clinical information are available online at http://www.gpecimage.ubc.ca (username: BCCA-VGH; password: OVCARE). The rate of positive cases for each biomarker ranged from 9% (KISS1) to 83% (EpCam) (detailed expression rates are listed in Table S2). Comparing biomarker expression in the entire cohort for tumors diagnosed at different stages revealed that ten biomarkers (CRABP-II, ER, F-Spondin, K-Cadherin, Ki-67, Matriptase, Mesothelin, p21, p53, and WT1) had significantly different expression levels between stages, suggesting differences between “early” and “late” stage disease (Figure 2, Table S2). However, comparing biomarker expression within one subtype across FIGO stages, no biomarker remained significantly differently expressed by stage (results for high-grade serous subtype are shown in Figure 3). This result was true for all four major subtypes (unpublished data for endometrioid, clear cell, and mucinous). In contrast, 20 of 21 biomarkers were significantly differentially expressed between the subtypes (Figure 4). Only, EpCam (*p* = 0.23) showed a consistent expression frequency across all subtypes. Additionally, *p*-values for biomarker expression rates in the entire cohort across subtypes were generally smaller than across stages (Table S2), indicating a stronger association with subtype than stage.

**Figure 5:**
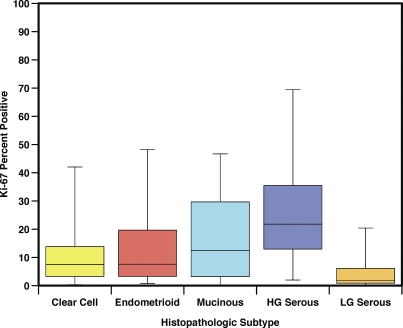
Distribution of Ki-67 Labelling Index across Subtypes

High-grade serous carcinoma showed positive staining in >75% of cases for WT1, Mesothelin, ER, and CA125 (Table S2). The biomarker expression pattern of low-grade serous carcinomas was similar to that of their high-grade counterparts. Three markers (PR, p53, K-Cadherin) showed a trend towards differential expression in low-grade versus high-grade serous subtypes. Only the median Ki-67 labelling index differed significantly between those groups, with median Ki-67 labelling index of 2.5% (95% confidence interval [CI] 0.5%–20.4%) in low-grade serous versus 22.4% (95% CI 3.6%–69.9%) in high-grade serous subtype (Figure 5). Endometrioid carcinomas coexpress high rates of hormone receptors ER and PR as well as CA125. Endometrioid and clear cell subtypes infrequently (<10%) expressed WT1 and p53. The median Ki-67 labelling index for endometrioid and clear cell carcinomas was similar (endometrioid 8.2%, 95% CI 0.8%–49.0%; clear cell 7.6%, 95% CI 0.5%–45.0%). Immunophenotypic characteristics of clear cell carcinomas included low levels of hormone receptors ER (10%) and PR (3%). The mucinous subtype displayed an intermediate proliferative capacity compared with the other subtypes (median Ki-67 labelling index 12.9%, 95% CI 2.1%–60.9%) and frequent expression of Matriptase (86%). Many of the markers expressed in other subtypes were either infrequently (<10%) expressed (p53, ER, PAX8, SLPI, K-Cadherin, and CA125), or completely absent (CRABP2, WT-1, and Mesothelin). Of note, EpCam was highly expressed across all subtypes included in this study.

### Survival Analyses Can Be Confounded by Subtype Differences

To assess the biological importance of a biomarker, its expression is usually correlated with outcome. Survival analysis was restricted to the three major subtypes (high-grade serous, clear cell, and endometrioid) because of insufficient numbers of cases of mucinous or low-grade serous subtypes. The primary endpoint was defined as DSS and the rates after 10 y are shown for subtypes in Table 1. A multivariable Cox regression model including age, stage, and histological subtype showed significant differences across stage (*p* < 0.0001) and subtype (*p* = 0.015). Survival by stage showed little difference between stages I and II, with stage III showing poorer DSS (RR 3.0, 95% CI 1.87%–4.66% relative to stage I). Survival by subtype showed poorer DSS for clear cell (RR 2.31, 95% CI 1.29%–4.15%) and high-grade serous (RR 2.74, 95% CI 1.56%–4.81%) relative to endometrioid subtype. Age was not predictive in the model (*p* = 0.211) (Table S3).

**Figure 6:**
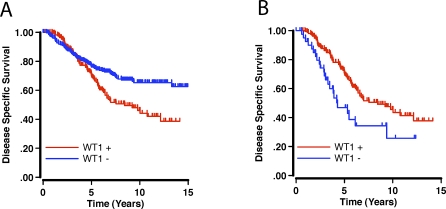
Prognostic Associations of WT1 Kaplan-Meier survival analysis of DSS. (A) Entire cohort grouped by WT1 positive versus negative cases (*p* = 0.0017, univariable COX regression). (B) high-grade serous subtype grouped by WT1 positive versus negative cases (*p* = 0.0086, univariable COX regression).

Univariable Cox regression analysis for each biomarker was applied on the entire cohort as well as within the three largest subtypes (Figure S1, Table 3). RRs and *p*-values are presented in Table 3. Nine of 21 biomarkers show prognostic significance in the entire cohort. Of the nine biomarkers showing a significant association with DSS in the entire cohort, three remain prognostic indicators in the high-grade serous and one in the endometrioid subtype. As an extreme example, WT1 is an unfavourable prognostic biomarker in the entire cohort (*p* = 0.0017, Figure 6A) but is a favourable prognostic biomarker for high-grade serous carcinomas (*p* = 0.0086, Figure 6B). As WT1 is expressed in 80% of high-grade serous carcinomas but rarely in other subtypes, this negative prognostic significance in the entire cohort reflects subtype differences in expression, with WT1 most commonly expressed in the aggressive high-grade serous subtype. Four other biomarkers (KISS1, K-Cadherin, Mesothelin, Ki-67) that were significant in the entire cohort did not show significance in any subtype.

**Table 3:**
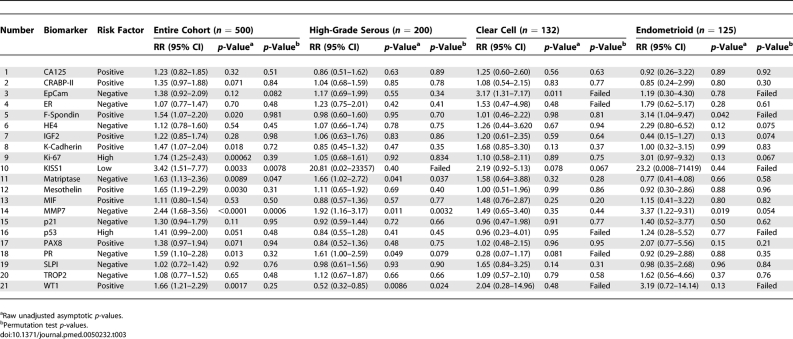
Univariable COX Regression for Disease-Specific Survival

Ki-67 serves as an additional example, which is prognostic in the whole cohort but not when corrected for subtype. The median for Ki-67 labelling index in the entire cohort was 13.0% and using this as a cut-off for high versus low Ki-67 labelling index effectively separates high-grade serous carcinomas from low-grade serous, endometrioid, and clear cell carcinomas (Figure 5). Mucinous carcinomas showed an intermediate Ki-67 labelling index. Associated with high-grade serous subtype, it is not surprising that Ki-67 has prognostic relevance in the whole cohort (*p* = 0.0062). When using the subtype specific median for separate analysis of each subtype however, Ki-67 labelling index was not of prognostic significance in any of the subtypes but Ki-67 labelling index was different between subtypes.

## Discussion

Ovarian carcinomas subtypes are associated with distinct genetic risk factors, underlying molecular events during oncogenesis, stage at diagnosis, and responses to chemotherapy. With slight modification of the WHO criteria for histopathological assignment for subtype we have recently shown that classification of ovarian carcinomas into five subtypes (high-grade serous, low-grade serous, clear cell, endometrioid, and mucinous) is reproducible and is supported by biomarker expression data [5].

By demonstrating that biomarker correlations with stage or prognosis can be explained by variations in expression rates between subtypes, our study offers persuasive evidence supporting the view that ovarian carcinoma subtypes are different diseases. Biomarker expression is stable across stage within a given subtype. Furthermore, differences in the expression profile between subtypes confound survival analysis for biomarkers, when multiple ovarian carcinoma subtypes are considered together. Collectively, these data have implications for ovarian carcinoma research and treatment.

Cancer treatment in general is beginning to move towards therapies tailored for specific cancer subtypes (e.g., breast carcinoma and lymphoma [24,25]), and this subtype specific approach to treatment has implications for the design of clinical trials for women with ovarian carcinomas. It has been recognized for some time that certain ovarian carcinoma subtypes are less sensitive to platinum-based chemotherapy than the most common high-grade serous carcinomas. The clear cell and mucinous subtypes, in particular, are candidates for clinical trials to identify more active therapy than what is currently used [9]. Given the dramatic differences in biomarker expression between ovarian carcinoma subtypes, our analysis suggests that advancing our understanding of these poorly understood subtypes—including identification of potential therapeutic targets—will only come through studies focusing on these specific subtypes rather than studies of unselected series of patients.

The biomarker expression profile within a given subtype is consistent across stage. Hence, early and advanced stage ovarian carcinomas differ primarily based on subtype, while within a subtype there is no difference between early and advanced stage tumors. This distinction has implications for the research on biomarkers for ovarian carcinoma screening, where the goal is detection of early stage disease, which has a much greater likelihood of cure. If subtypes are neglected, a screening marker identified in advanced stage tumors (i.e., high-grade serous carcinomas), may not be expressed in most nonserous early stage ovarian carcinomas, and vice versa. For example, CA125 is expressed in most high-grade serous carcinoma, but only in 60% of mucinous and clear cell subtypes, a finding that is consistent with previous studies [26]. A related observation is that serum CA125 levels are elevated in 80% of patients with advanced stage epithelial ovarian carcinoma but are increased in only 60% of patients with early stage disease [27,28]. It is likely that a panel of tumor markers will be required to detect all subtypes. As the biomarker expression was consistent between stages within the subtypes, these data support the use of late stage cancers to identify biomarkers for the early detection of cancers of the same subtype.

Biomarker correlation with prognosis can be confounded by subtype differences in biomarker expression. Some biomarkers show prognostic significance independent from subtype, e.g., we confirmed that MMP7 expression is a strong independent prognostic factor for favourable prognosis in the entire cohort, as shown previously [29] as well as for high-grade serous and endometrioid subtypes. This result is the exception rather than the rule as for most of the biomarkers, the correlation with prognosis in the entire cohort is due to the correlation with the most common subtype (high-grade serous carcinoma), which in turn is associated with a poor prognosis. The biomarkers that were of prognostic significance in subtype analysis were typically only of prognostic value for a single subtype. WT1 is a widely used diagnostic marker for the serous subtype [30] and is an example for how analysis of the entire cohort can give misleading results. In the entire cohort WT1 is an unfavourable prognostic marker but is a favourable prognostic marker for high-grade serous tumors (Figure 6). This latter observation may be because WT1 is a marker for serous differentiation, and less differentiated high-grade serous cancers are both less likely to express WT1 and have a worse prognosis. This inverse association in a subgroup, also known as Simpson's paradox, will not typically be revealed by multivariable analysis [19].

Another example is Ki-67; there are conflicting results on the prognostic value of Ki-67 in ovarian carcinoma [31–36]. After applying a single cut-off point on the entire cohort for identification of Ki-67 high and Ki-67 low cases, high Ki-67 index is associated with an unfavourable prognosis. But differences in Ki-67 indices between subtypes again confound the analysis because nearly all high-grade serous carcinomas have a high Ki-67 index. In analysis by subtype, Ki-67 is not of prognostic significance; the effect seen in the entire cohort reflects an association with the high-grade serous subtype.

Adjustment for multiple comparisons is an important consideration. However, there are several bodies of data under discussion in different sections of this report, with differing numbers of comparisons. For example, assessing the proportion of positive cases across histological subtypes for each biomarker involves the assessment of 21 tests; whereas assessing survival within FIGO and histological subtype groups involves more tests. Since *p*-value adjustment for multiple testing uses the number of tests under consideration, several collections of adjusted *p*-values would have to be constructed yielding a complex distraction from the discussion at hand. We note that for the assessment of proportion of positive cases, the Bonferroni-adjusted level of significance would be 0.05/21 = 0.0024, and several *p*-values in that analysis are less than this level. We report raw *p*-values so that the reader can apply multiple comparison adjustments relevant to the size of comparisons being made in any section of the paper, and in future meta-analyses of subsets of these data. Corrections for multiple comparisons were not used because this issue of prognostic significance is not the central theme of the manuscript. Prognostic significance is used to illustrate the importance of subtype-specific analysis. A limitation of our study is that it is performed retrospectively [37], and 94% of patients received adjuvant platinum-based chemotherapy. Hence, we can not adhere to the strict definition of a prognostic marker as applying only to the natural history of the disease.

We hope these data will end the lumping of ovarian carcinoma subtypes within biomarker studies, as is the current practice [38–41]. This biomarker panel shows that subtypes have distinct expression profiles. One of the reasons why no ovarian carcinoma tissue-based prognostic markers are used clinically, despite a voluminous literature suggesting many candidates, is that prognostic effects have proven difficult to validate. In addition to assay specific challenges, the different frequencies of subtypes within cohorts can vary or, as shown here with WT1, reverse prognostic effects. If ovarian carcinoma cases are not separated by subtype or evaluated using a stratified analysis or a model with complex interaction terms, even a multivariable model can conceal important findings or lead to misleading conclusions (Table S4). The discovery, development, and validation of subtype specific ovarian carcinoma biomarkers will require adequately powered and expertly subtyped cohorts of cases. For the rarer subtypes, the development of such research resources will likely prove difficult outside of large scale collaborative initiatives. In order to facilitate the shift to subtype specific management of ovarian carcinoma, subtypes should be considered as distinct diseases in biomarker studies and clinical trials.

## Supporting Information

Figure S1 Kaplan-Meier Survival Analysis of Disease-Specific Survival in the Entire Cohort, Stage Subgroups, Subtypes, and the Stage Subgroups by Subtype *p*-Values (Wald) were generated using a multivariable Cox regression model including the biomarker and age. “Marker * CellType Xn *p*”-value assesses differential biomarker prognostic value in the different subtypes (a large *p*-value indicates that biomarker prognosis is similar in the subgroups, a small *p*-value indicates that biomarker prognosis differs in the subgroups); “Marker * FIGO Xn *p*”-value assesses differential biomarker prognostic value in the different subgroups. HG-SC, high-grade serous; EC, endometrioid; CC, clear cell; FIGO=stg12, FIGO stage I and II; FIGO=stg3, FIGO stage III.
(2 MB PDF)

Table S1 Definition of Positive Staining (42 KB DOC)

Table S2 Biomarker Expression Rate Across Stage, Subtype, and Stage within Subtypes (133 KB DOC)

Table S3 Multivariable COX Proportional Hazards Including Stage, Subtype, and Age for the Entire Cohort (30 KB DOC)

Table S4 Multivariable COX Proportional Hazards Including Stage, Subtype, Age, and WT-1 for the Entire Cohort and High-Grade Serous Carcinomas (38 KB DOC)

## Abbreviations

BCCA: British Columbia Cancer Agency
CI: confidence interval
DSS: disease-specific survival
RR: risk ratio
TMA: tissue microarray
