# Supplementary material for: Ovarian Carcinoma Subtypes Are Different Diseases: Implications for Biomarker Studies
Source: PLoS Med. 2008 Dec 2;5(12):e232. doi: 10.1371/journal.pmed.0050232 (PMC2592352; doi:10.1371/journal.pmed.0050232)
Supplement: Supplementary file 1 [file pmed.0050232.sg001.pdf]

Biomarker = CA125 (Age−adjusted p−values shown)

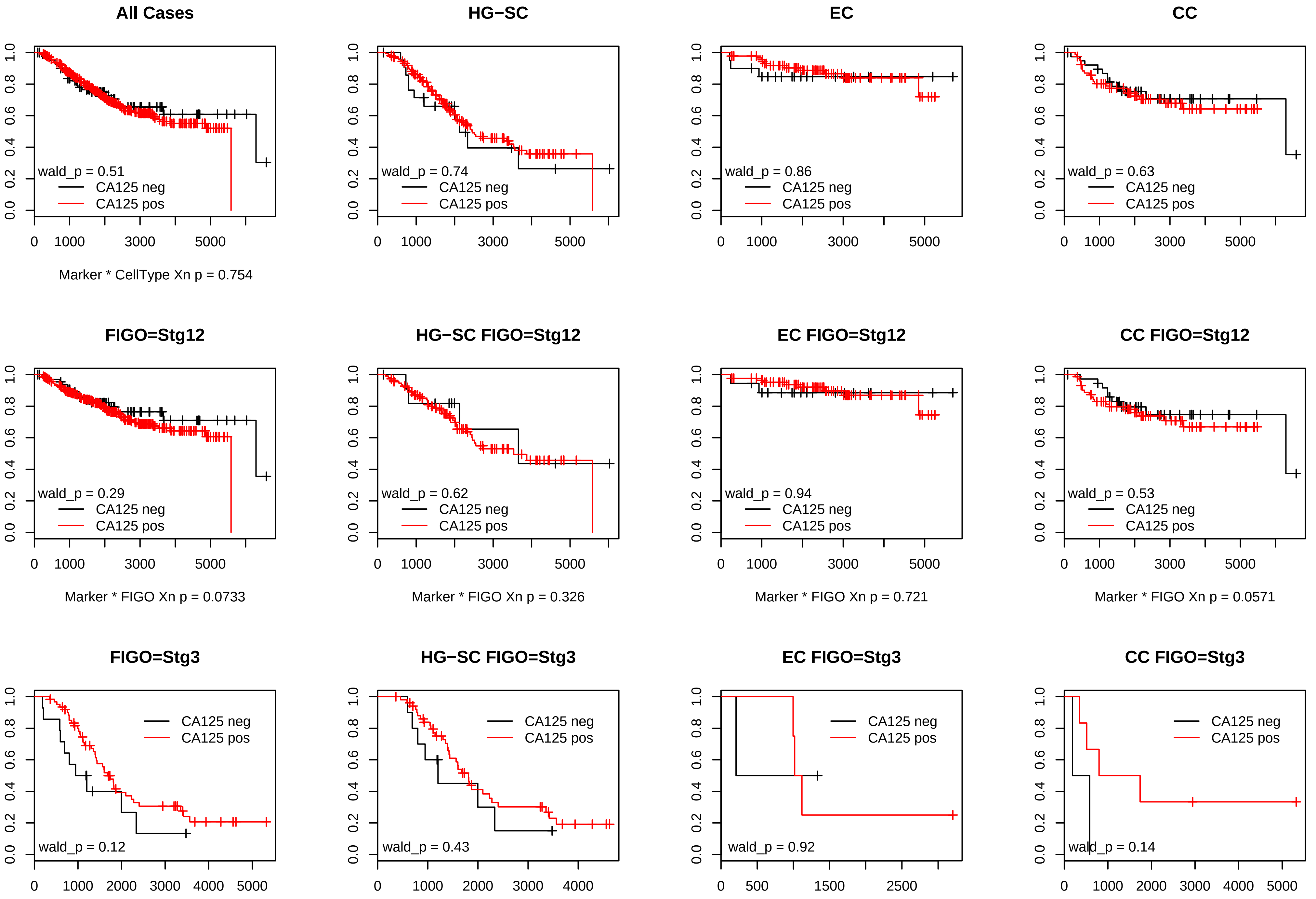

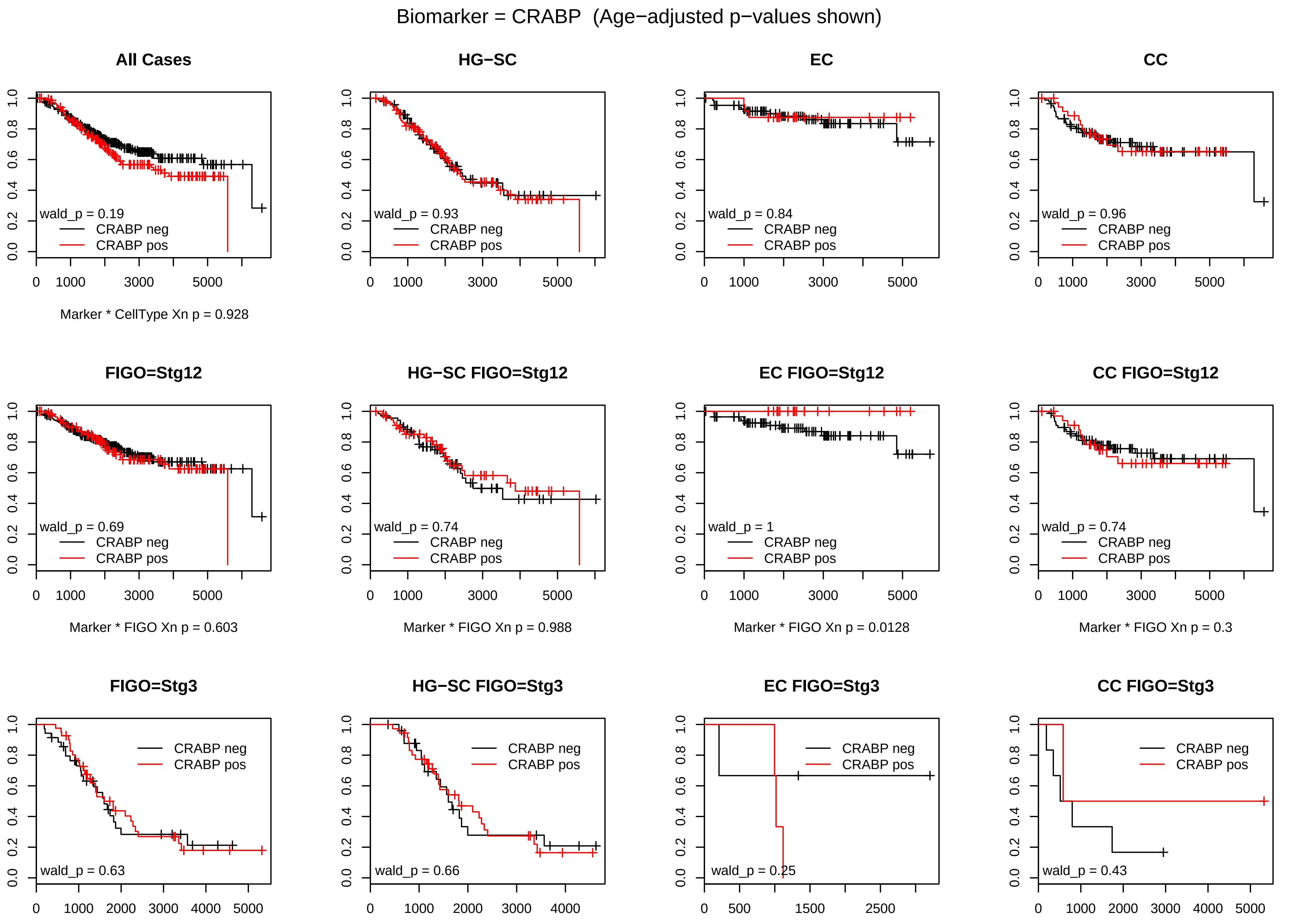
Biomarker = CRABP (Age−adjusted p−values shown)
All Cases
wald_p = 0.19
CRABP neg
CRABP pos
Marker * CellType Xn p = 0.928
HG−SC
wald_p = 0.93
EC
wald_p = 0.84
CC
wald_p = 0.96
FIGO=Stg12
wald_p = 0.69
Marker * FIGO Xn p = 0.603
HG−SC FIGO=Stg12
wald_p = 0.74
Marker * FIGO Xn p = 0.988
EC FIGO=Stg12
wald_p = 1
Marker * FIGO Xn p = 0.0128
CC FIGO=Stg12
wald_p = 0.74
Marker * FIGO Xn p = 0.3
FIGO=Stg3
wald_p = 0.63
HG−SC FIGO=Stg3
wald_p = 0.66
EC FIGO=Stg3
wald_p = 0.25
CC FIGO=Stg3
wald_p = 0.43

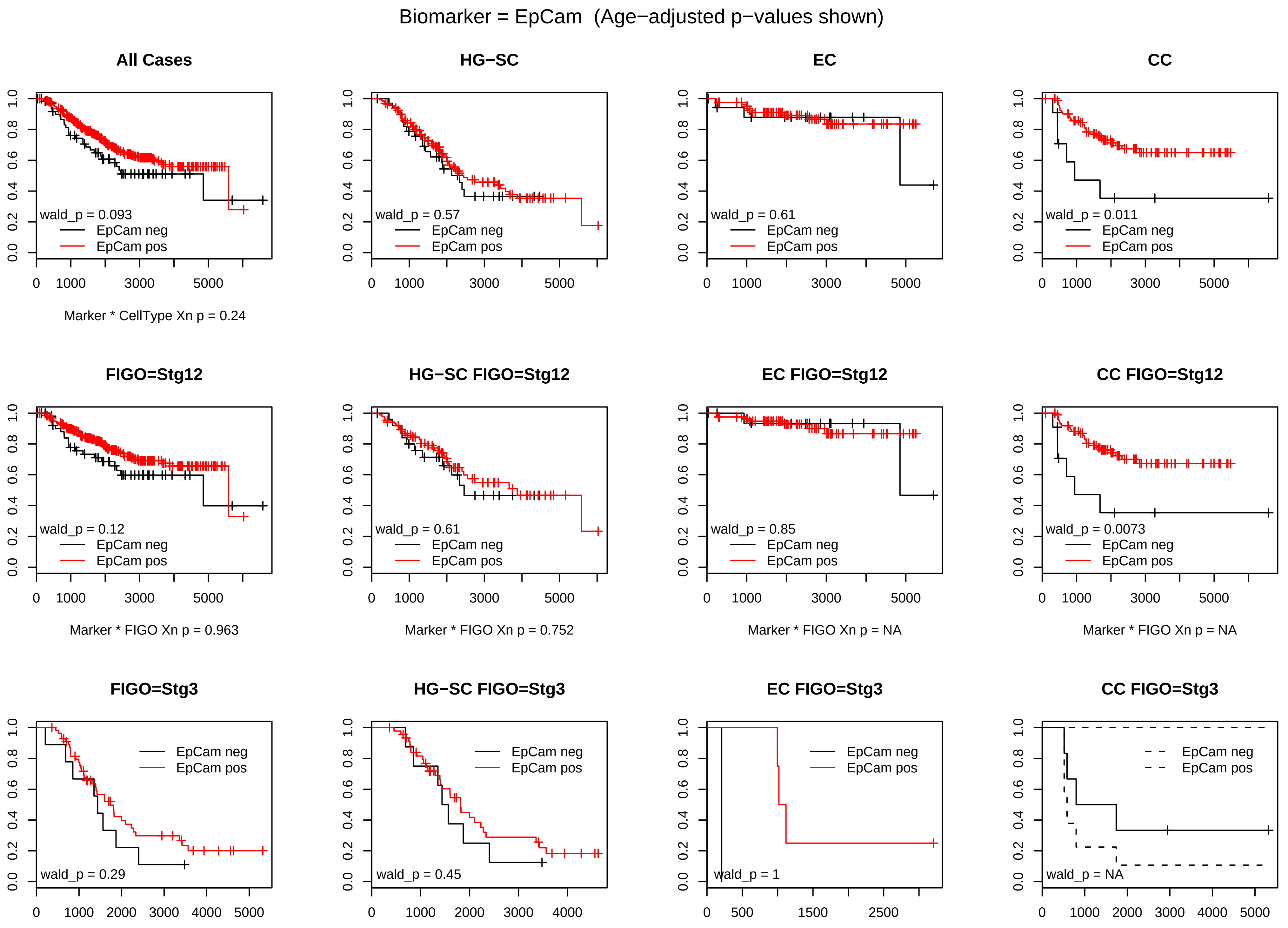

Biomarker = EpCam (Age−adjusted p−values shown)
All Cases
wald_p = 0.093
EpCam neg
EpCam pos
Marker * CellType Xn p = 0.24
HG−SC
wald_p = 0.57
EpCam neg
EpCam pos
EC
wald_p = 0.61
EpCam neg
EpCam pos
CC
wald_p = 0.011
EpCam neg
EpCam pos
FIGO=Stg12
wald_p = 0.12
EpCam neg
EpCam pos
Marker * FIGO Xn p = 0.963
HG−SC FIGO=Stg12
wald_p = 0.61
EpCam neg
EpCam pos
Marker * FIGO Xn p = 0.752
EC FIGO=Stg12
wald_p = 0.85
EpCam neg
EpCam pos
Marker * FIGO Xn p = NA
CC FIGO=Stg12
wald_p = 0.0073
EpCam neg
EpCam pos
Marker * FIGO Xn p = NA
FIGO=Stg3
EpCam neg
EpCam pos
wald_p = 0.29
HG−SC FIGO=Stg3
EpCam neg
EpCam pos
wald_p = 0.45
EC FIGO=Stg3
EpCam neg
EpCam pos
wald_p = 1
CC FIGO=Stg3
EpCam neg
EpCam pos
wald_p = NA

Biomarker = ER (Age−adjusted p−values shown)

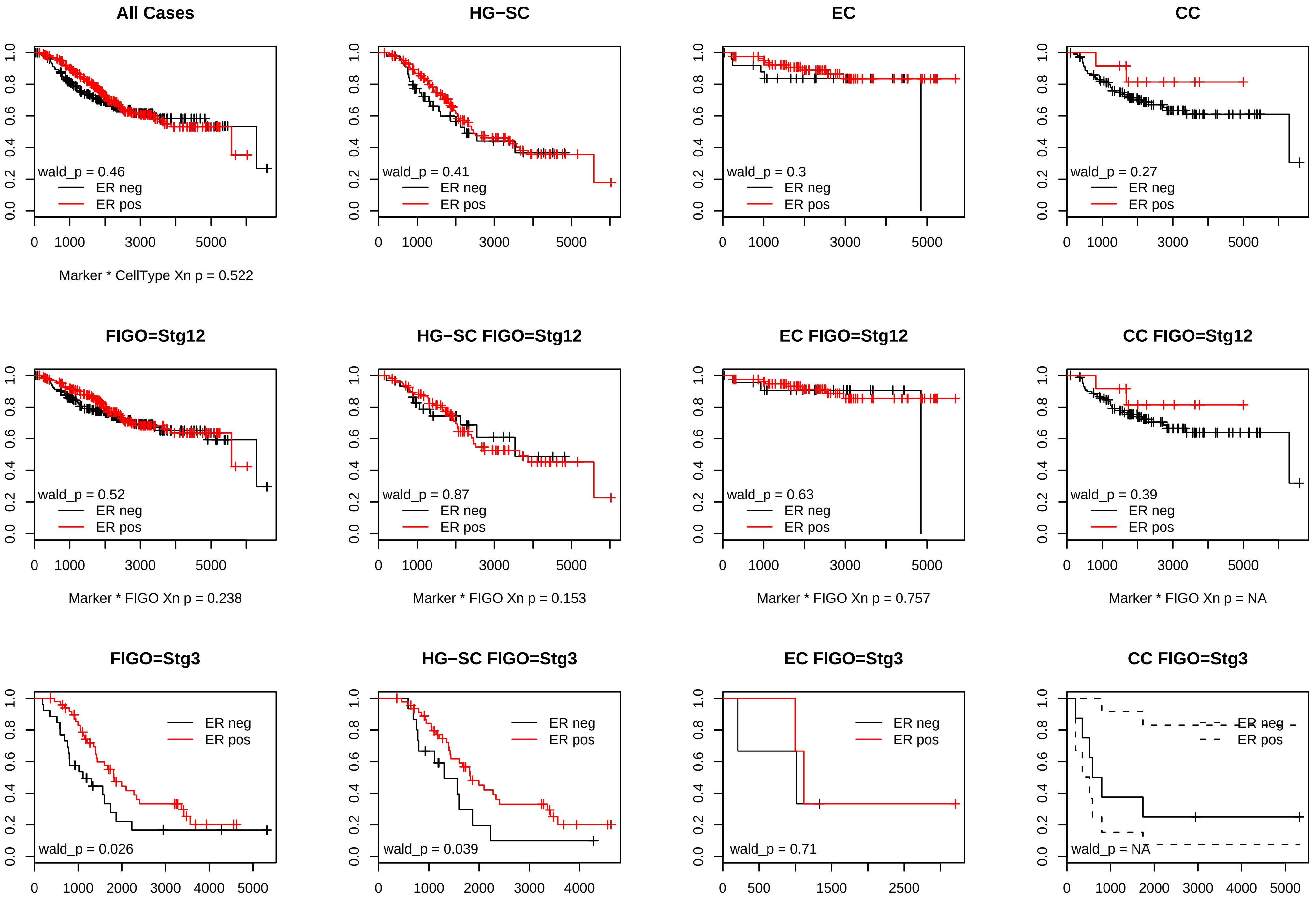

Biomarker = F_Spondin (Age−adjusted p−values shown)

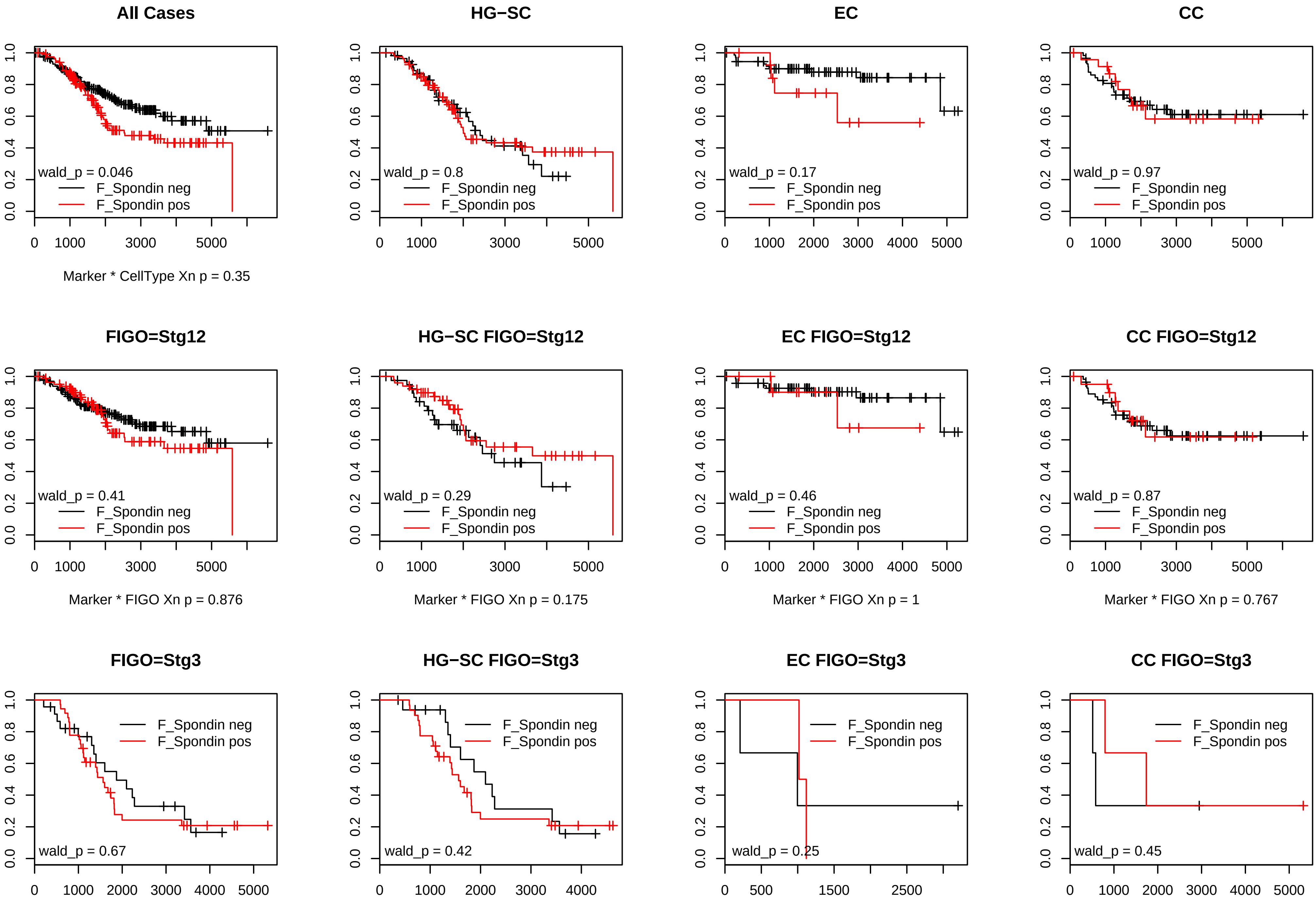

Biomarker = HE4 (Age−adjusted p−values shown)

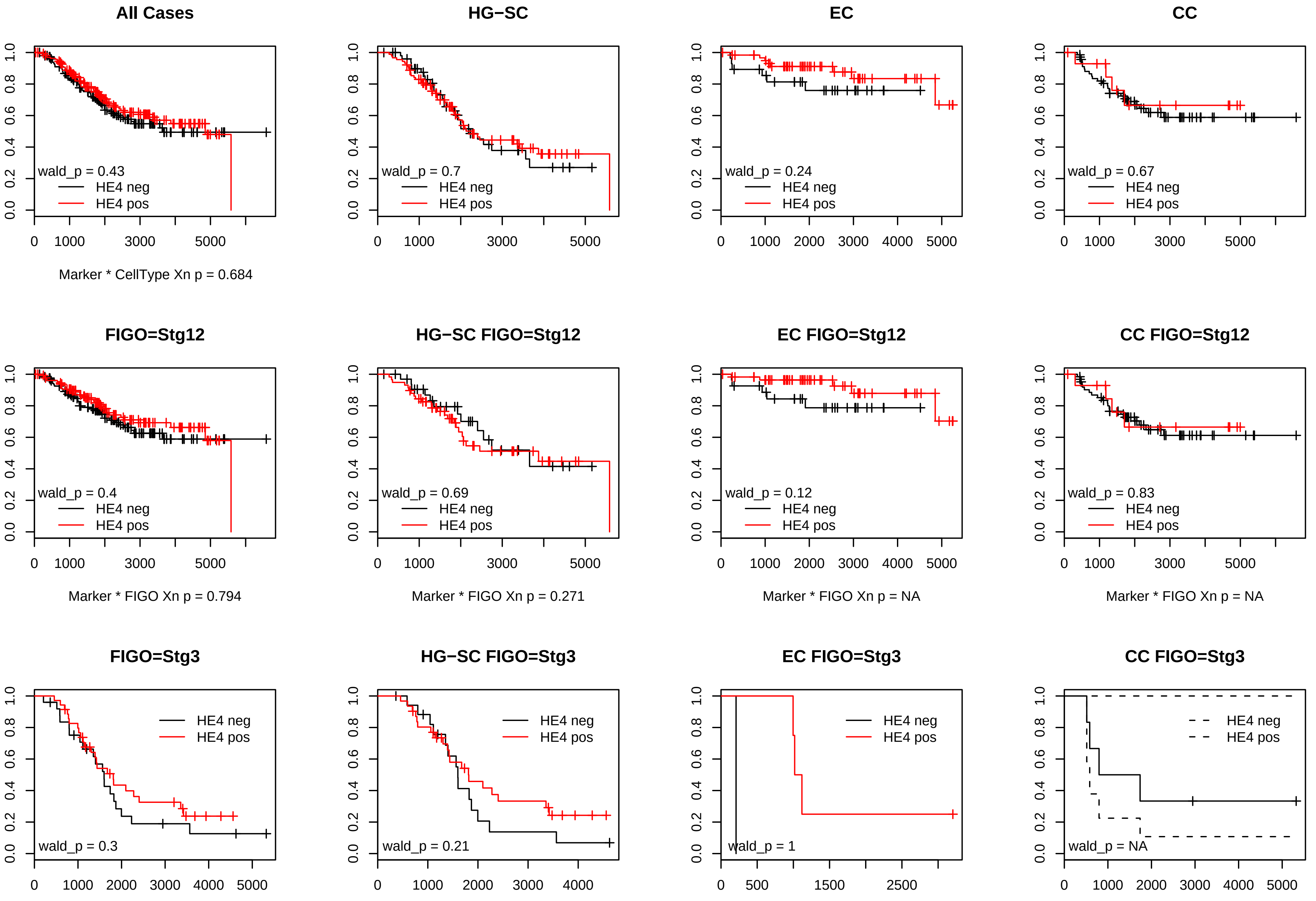

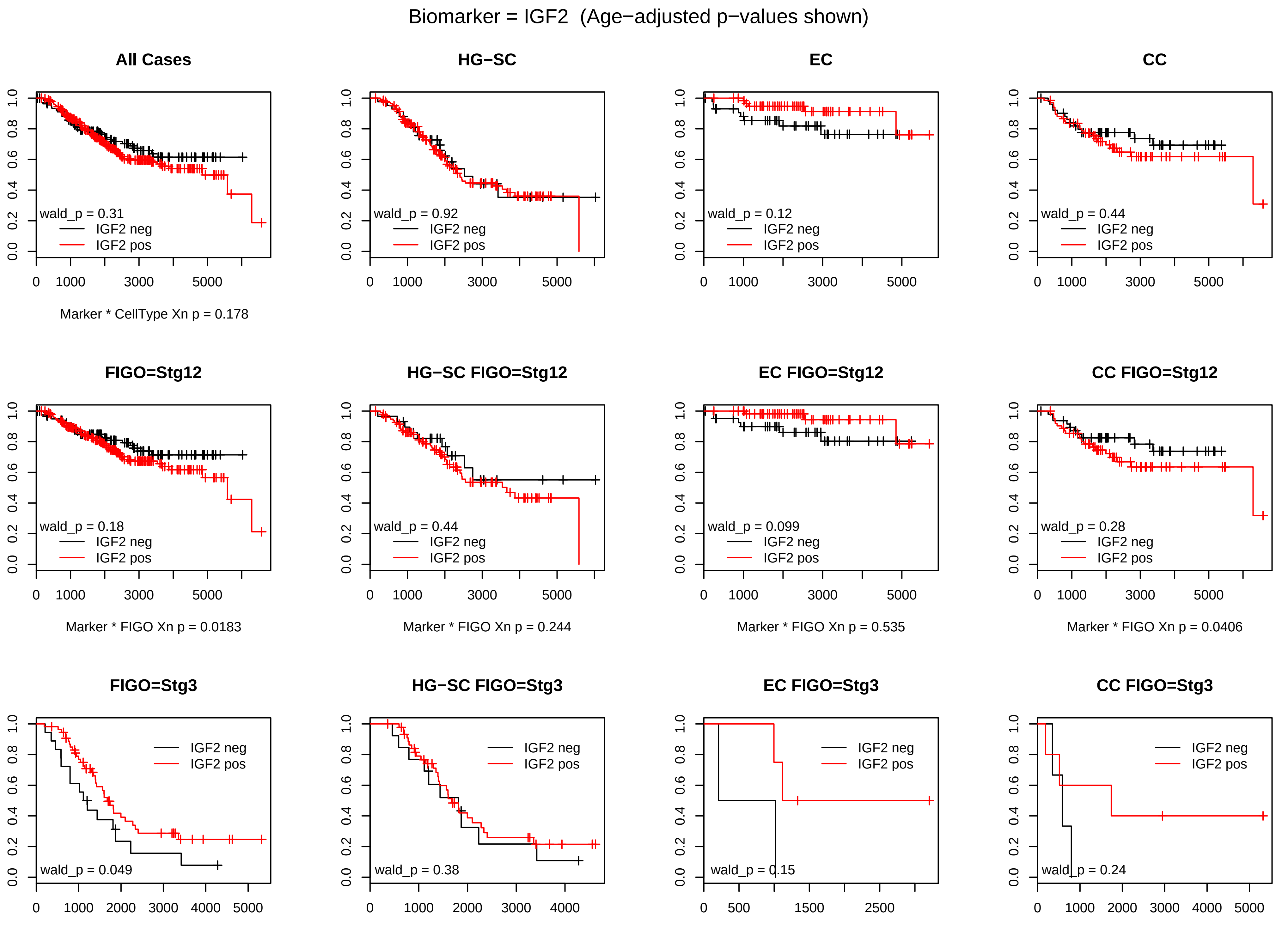
Biomarker = IGF2 (Age−adjusted p−values shown)
All Cases
wald_p = 0.31
IGF2 neg
IGF2 pos
Marker * CellType Xn p = 0.178
HG−SC
wald_p = 0.92
EC
wald_p = 0.12
CC
wald_p = 0.44
FIGO=Stg12
wald_p = 0.18
Marker * FIGO Xn p = 0.0183
HG−SC FIGO=Stg12
wald_p = 0.44
Marker * FIGO Xn p = 0.244
EC FIGO=Stg12
wald_p = 0.099
Marker * FIGO Xn p = 0.535
CC FIGO=Stg12
wald_p = 0.28
Marker * FIGO Xn p = 0.0406
FIGO=Stg3
wald_p = 0.049
HG−SC FIGO=Stg3
wald_p = 0.38
EC FIGO=Stg3
wald_p = 0.15
CC FIGO=Stg3
wald_p = 0.24

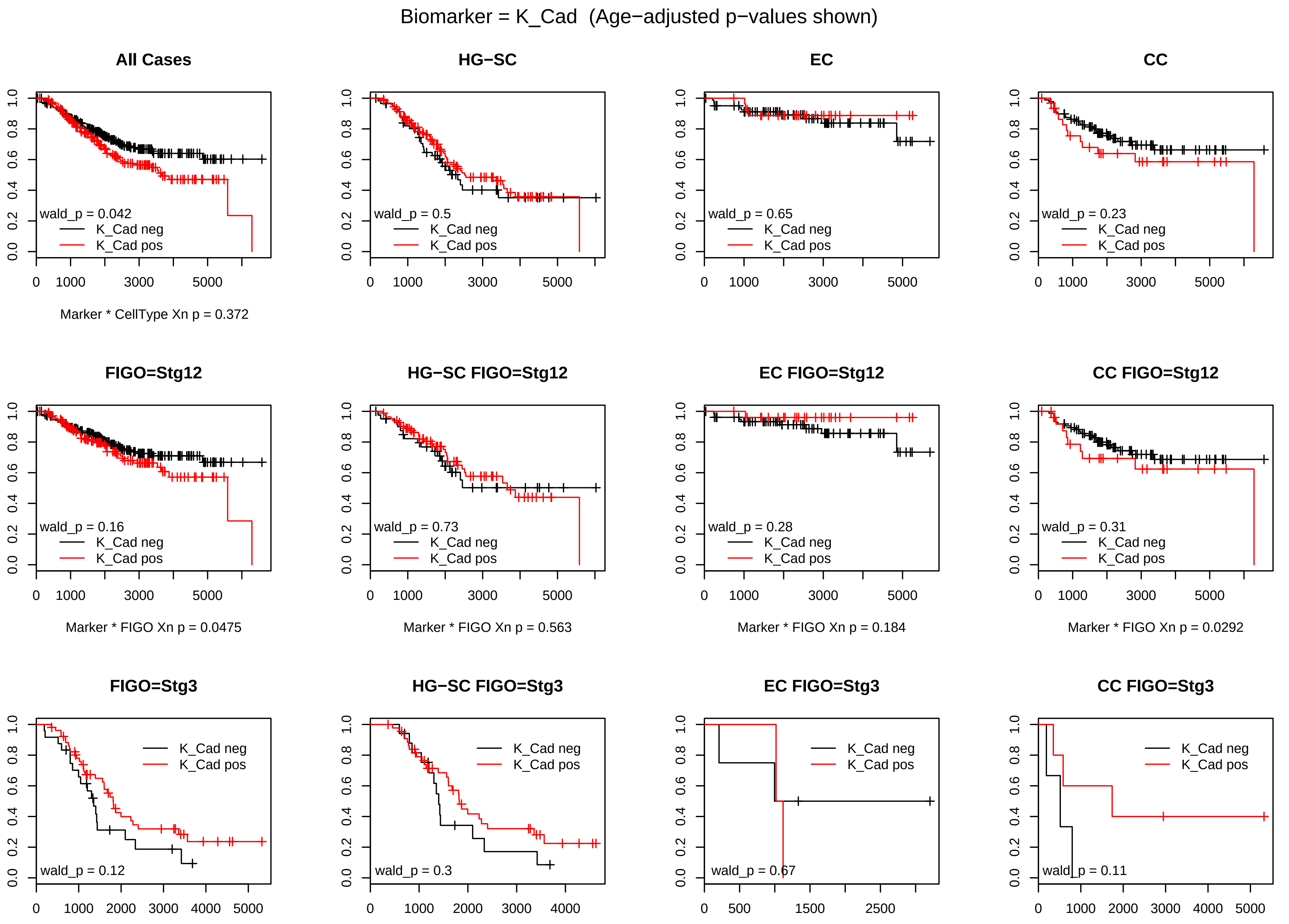

Biomarker = K_Cad (Age−adjusted p−values shown)
All Cases
wald_p = 0.042
K_Cad neg
K_Cad pos
Marker * CellType Xn p = 0.372
HG−SC
wald_p = 0.5
K_Cad neg
K_Cad pos
EC
wald_p = 0.65
K_Cad neg
K_Cad pos
CC
wald_p = 0.23
K_Cad neg
K_Cad pos
FIGO=Stg12
wald_p = 0.16
K_Cad neg
K_Cad pos
Marker * FIGO Xn p = 0.0475
HG−SC FIGO=Stg12
wald_p = 0.73
K_Cad neg
K_Cad pos
Marker * FIGO Xn p = 0.563
EC FIGO=Stg12
wald_p = 0.28
K_Cad neg
K_Cad pos
Marker * FIGO Xn p = 0.184
CC FIGO=Stg12
wald_p = 0.31
K_Cad neg
K_Cad pos
Marker * FIGO Xn p = 0.0292
FIGO=Stg3
K_Cad neg
K_Cad pos
wald_p = 0.12
HG−SC FIGO=Stg3
K_Cad neg
K_Cad pos
wald_p = 0.3
EC FIGO=Stg3
K_Cad neg
K_Cad pos
wald_p = 0.67
CC FIGO=Stg3
K_Cad neg
K_Cad pos
wald_p = 0.11

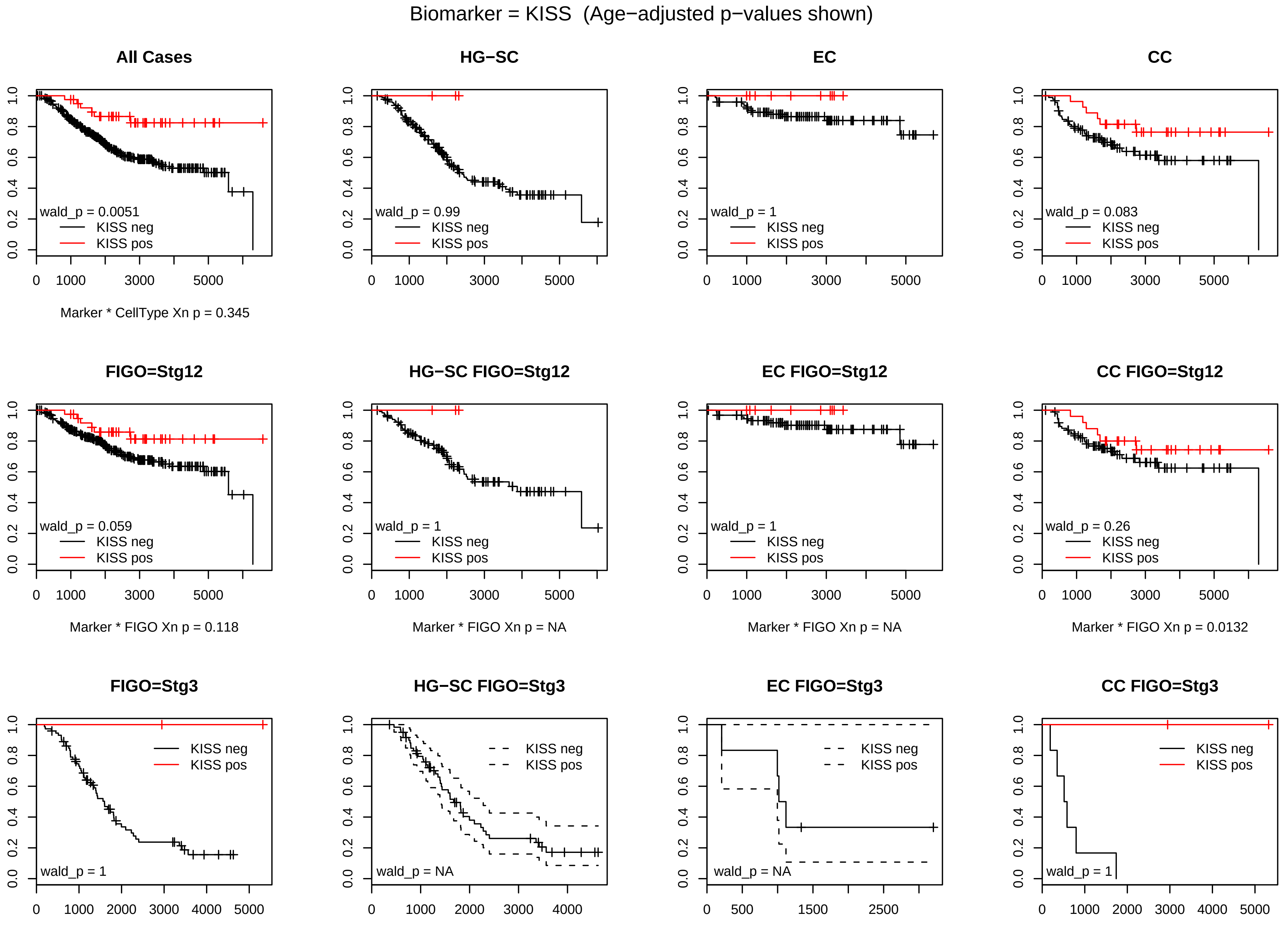

Biomarker = KISS (Age−adjusted p−values shown)
All Cases
wald_p = 0.0051
KISS neg
KISS pos
Marker * CellType Xn p = 0.345
HG−SC
wald_p = 0.99
KISS neg
KISS pos
EC
wald_p = 1
KISS neg
KISS pos
CC
wald_p = 0.083
KISS neg
KISS pos
FIGO=Stg12
wald_p = 0.059
KISS neg
KISS pos
Marker * FIGO Xn p = 0.118
HG−SC FIGO=Stg12
wald_p = 1
KISS neg
KISS pos
Marker * FIGO Xn p = NA
EC FIGO=Stg12
wald_p = 1
KISS neg
KISS pos
Marker * FIGO Xn p = NA
CC FIGO=Stg12
wald_p = 0.26
KISS neg
KISS pos
Marker * FIGO Xn p = 0.0132
FIGO=Stg3
KISS neg
KISS pos
wald_p = 1
HG−SC FIGO=Stg3
KISS neg
KISS pos
wald_p = NA
EC FIGO=Stg3
KISS neg
KISS pos
wald_p = NA
CC FIGO=Stg3
KISS neg
KISS pos
wald_p = 1

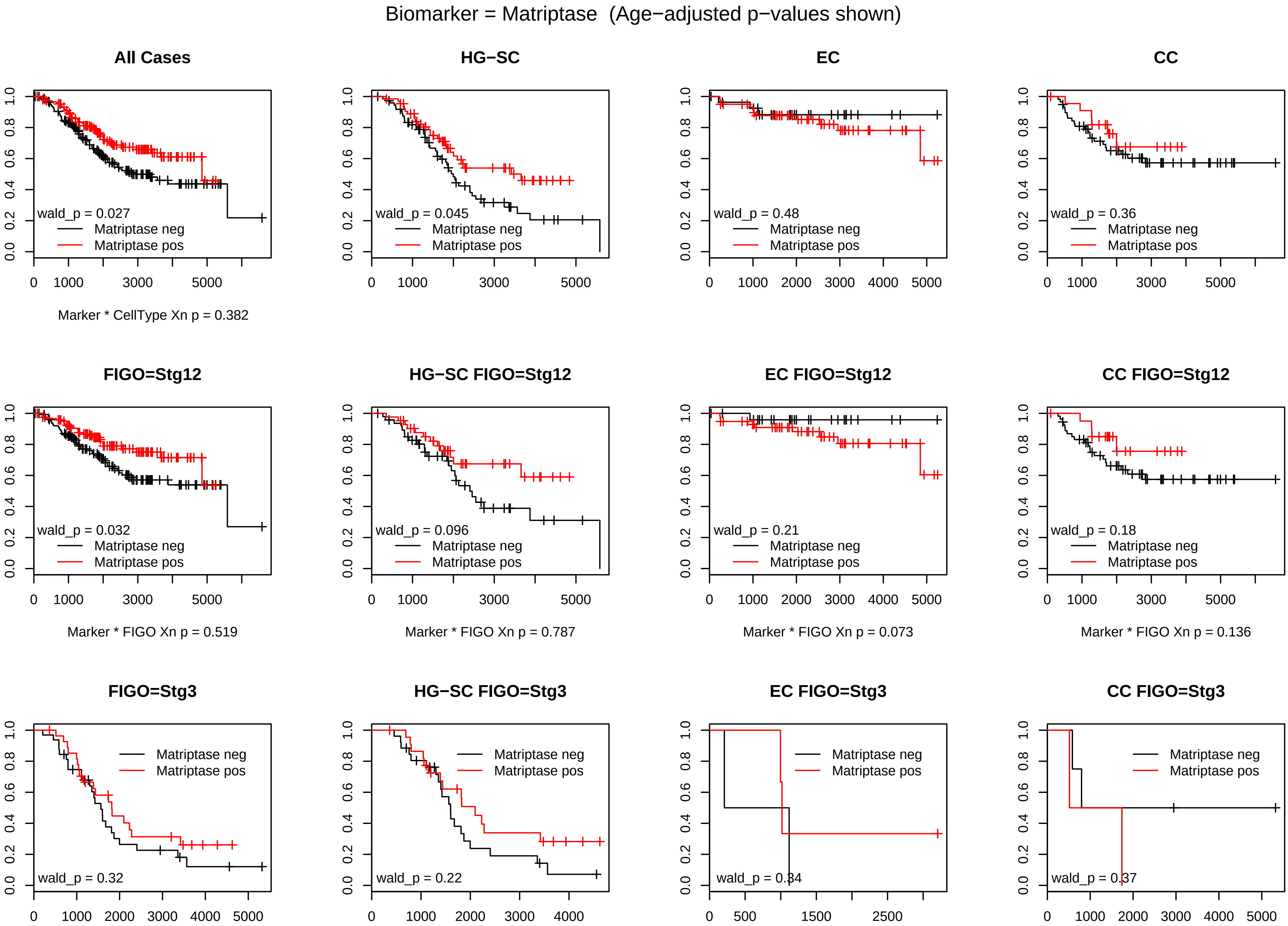
Biomarker = Matriptase (Age−adjusted p−values shown)
All Cases
wald_p = 0.027
Matriptase neg
Matriptase pos
Marker * CellType Xn p = 0.382
HG−SC
wald_p = 0.045
Matriptase neg
Matriptase pos
EC
wald_p = 0.48
Matriptase neg
Matriptase pos
CC
wald_p = 0.36
Matriptase neg
Matriptase pos
FIGO=Stg12
wald_p = 0.032
Matriptase neg
Matriptase pos
Marker * FIGO Xn p = 0.519
HG−SC FIGO=Stg12
wald_p = 0.096
Matriptase neg
Matriptase pos
Marker * FIGO Xn p = 0.787
EC FIGO=Stg12
wald_p = 0.21
Matriptase neg
Matriptase pos
Marker * FIGO Xn p = 0.073
CC FIGO=Stg12
wald_p = 0.18
Matriptase neg
Matriptase pos
Marker * FIGO Xn p = 0.136
FIGO=Stg3
Matriptase neg
Matriptase pos
wald_p = 0.32
HG−SC FIGO=Stg3
Matriptase neg
Matriptase pos
wald_p = 0.22
EC FIGO=Stg3
Matriptase neg
Matriptase pos
wald_p = 0.34
CC FIGO=Stg3
Matriptase neg
Matriptase pos
wald_p = 0.37

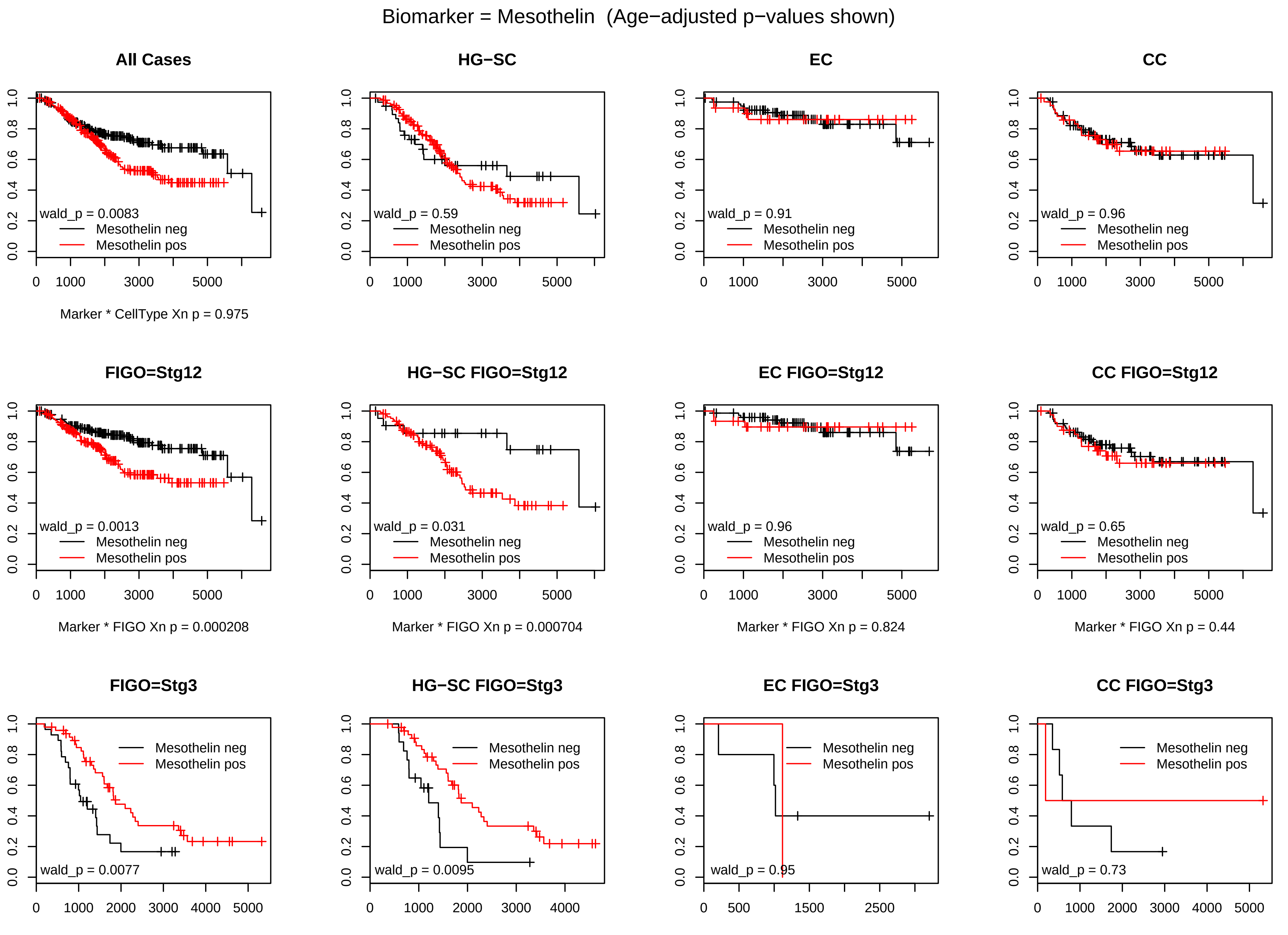
Biomarker = Mesothelin (Age−adjusted p−values shown)
All Cases
wald_p = 0.0083
Mesothelin neg
Mesothelin pos
Marker * CellType Xn p = 0.975
HG−SC
wald_p = 0.59
Mesothelin neg
Mesothelin pos
EC
wald_p = 0.91
Mesothelin neg
Mesothelin pos
CC
wald_p = 0.96
Mesothelin neg
Mesothelin pos
FIGO=Stg12
wald_p = 0.0013
Mesothelin neg
Mesothelin pos
Marker * FIGO Xn p = 0.000208
HG−SC FIGO=Stg12
wald_p = 0.031
Mesothelin neg
Mesothelin pos
Marker * FIGO Xn p = 0.000704
EC FIGO=Stg12
wald_p = 0.96
Mesothelin neg
Mesothelin pos
Marker * FIGO Xn p = 0.824
CC FIGO=Stg12
wald_p = 0.65
Mesothelin neg
Mesothelin pos
Marker * FIGO Xn p = 0.44
FIGO=Stg3
Mesothelin neg
Mesothelin pos
wald_p = 0.0077
HG−SC FIGO=Stg3
Mesothelin neg
Mesothelin pos
wald_p = 0.0095
EC FIGO=Stg3
Mesothelin neg
Mesothelin pos
wald_p = 0.95
CC FIGO=Stg3
Mesothelin neg
Mesothelin pos
wald_p = 0.73

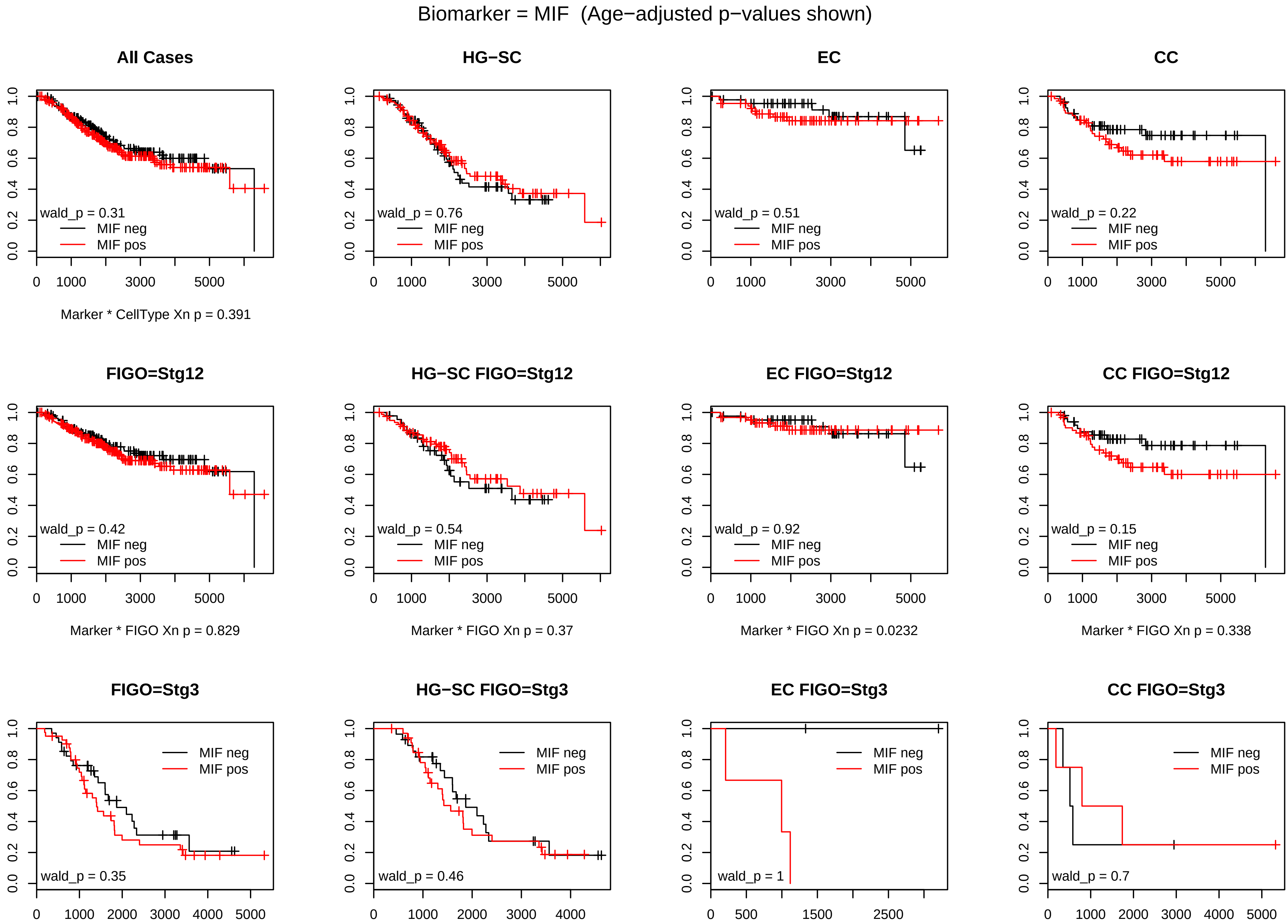
Biomarker = MIF (Age−adjusted p−values shown)
All Cases
wald_p = 0.31
MIF neg
MIF pos
Marker * CellType Xn p = 0.391
HG−SC
wald_p = 0.76
MIF neg
MIF pos
EC
wald_p = 0.51
MIF neg
MIF pos
CC
wald_p = 0.22
MIF neg
MIF pos
FIGO=Stg12
wald_p = 0.42
MIF neg
MIF pos
Marker * FIGO Xn p = 0.829
HG−SC FIGO=Stg12
wald_p = 0.54
MIF neg
MIF pos
Marker * FIGO Xn p = 0.37
EC FIGO=Stg12
wald_p = 0.92
MIF neg
MIF pos
Marker * FIGO Xn p = 0.0232
CC FIGO=Stg12
wald_p = 0.15
MIF neg
MIF pos
Marker * FIGO Xn p = 0.338
FIGO=Stg3
MIF neg
MIF pos
wald_p = 0.35
HG−SC FIGO=Stg3
MIF neg
MIF pos
wald_p = 0.46
EC FIGO=Stg3
MIF neg
MIF pos
wald_p = 1
CC FIGO=Stg3
MIF neg
MIF pos
wald_p = 0.7

Biomarker = MMP7 (Age−adjusted p−values shown)

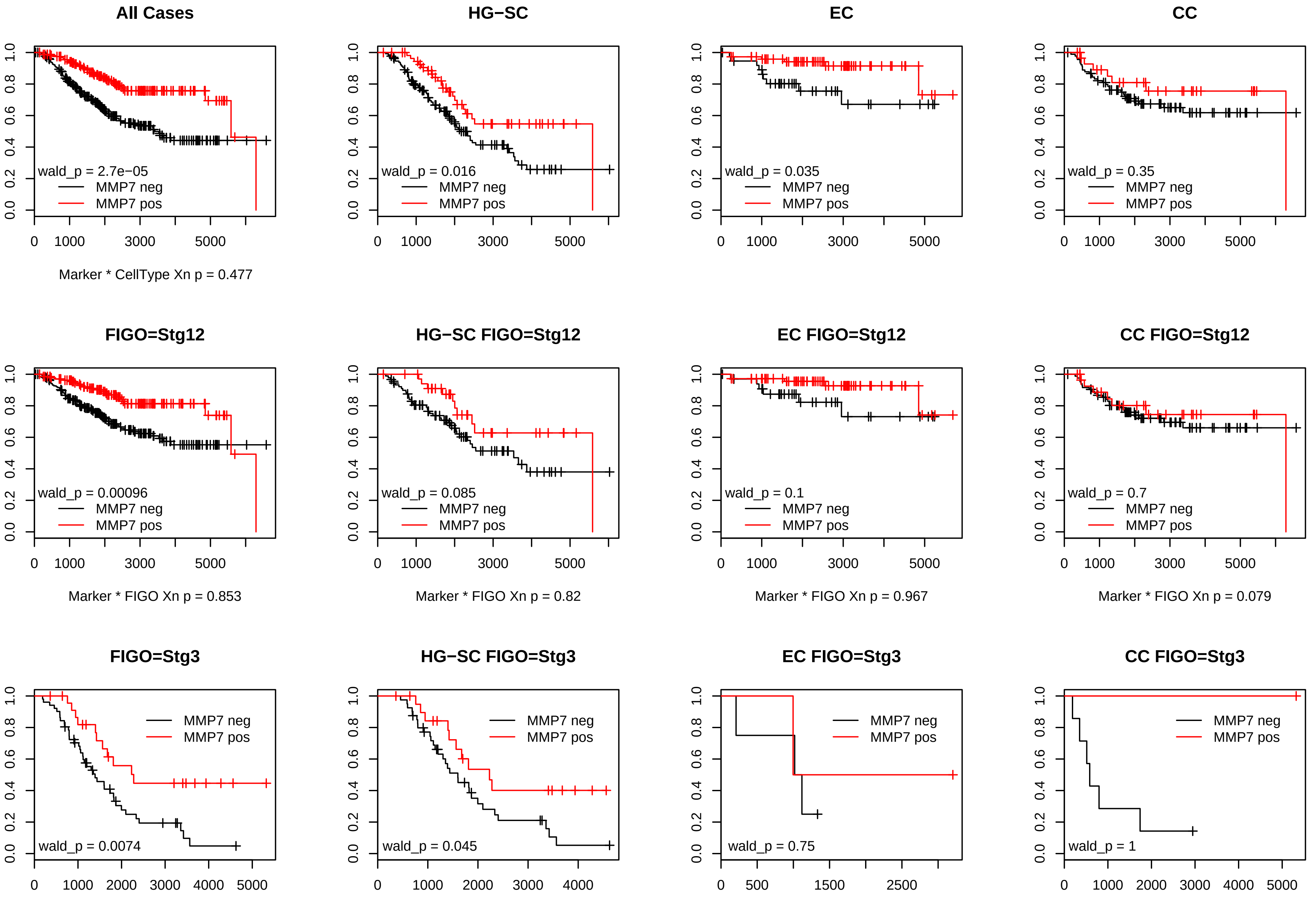

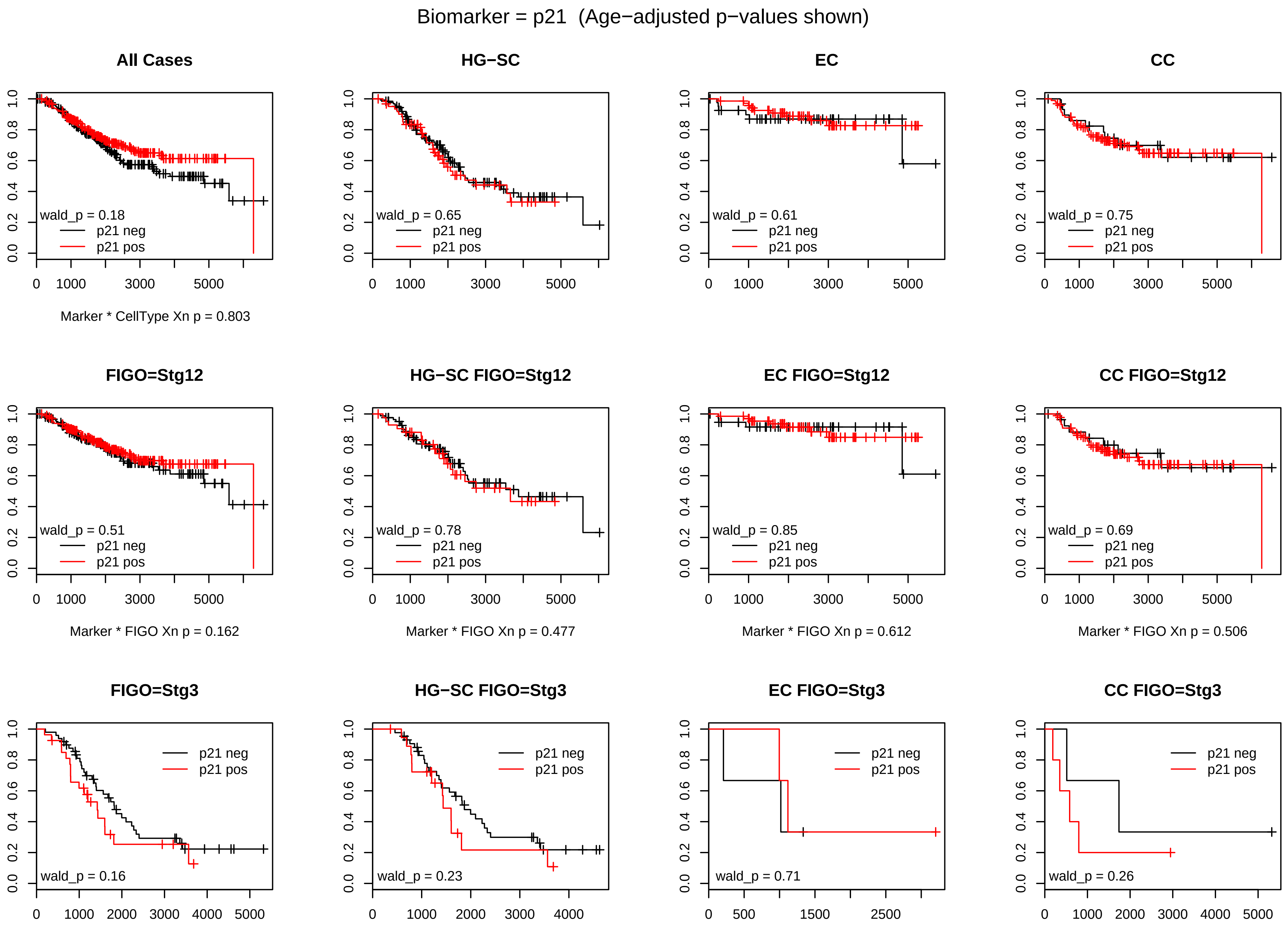

Biomarker = p21 (Age−adjusted p−values shown)
All Cases
wald_p = 0.18
p21 neg
p21 pos
Marker * CellType Xn p = 0.803
HG−SC
wald_p = 0.65
p21 neg
p21 pos
EC
wald_p = 0.61
p21 neg
p21 pos
CC
wald_p = 0.75
p21 neg
p21 pos
FIGO=Stg12
wald_p = 0.51
p21 neg
p21 pos
Marker * FIGO Xn p = 0.162
HG−SC FIGO=Stg12
wald_p = 0.78
p21 neg
p21 pos
Marker * FIGO Xn p = 0.477
EC FIGO=Stg12
wald_p = 0.85
p21 neg
p21 pos
Marker * FIGO Xn p = 0.612
CC FIGO=Stg12
wald_p = 0.69
p21 neg
p21 pos
Marker * FIGO Xn p = 0.506
FIGO=Stg3
p21 neg
p21 pos
wald_p = 0.16
HG−SC FIGO=Stg3
p21 neg
p21 pos
wald_p = 0.23
EC FIGO=Stg3
p21 neg
p21 pos
wald_p = 0.71
CC FIGO=Stg3
p21 neg
p21 pos
wald_p = 0.26

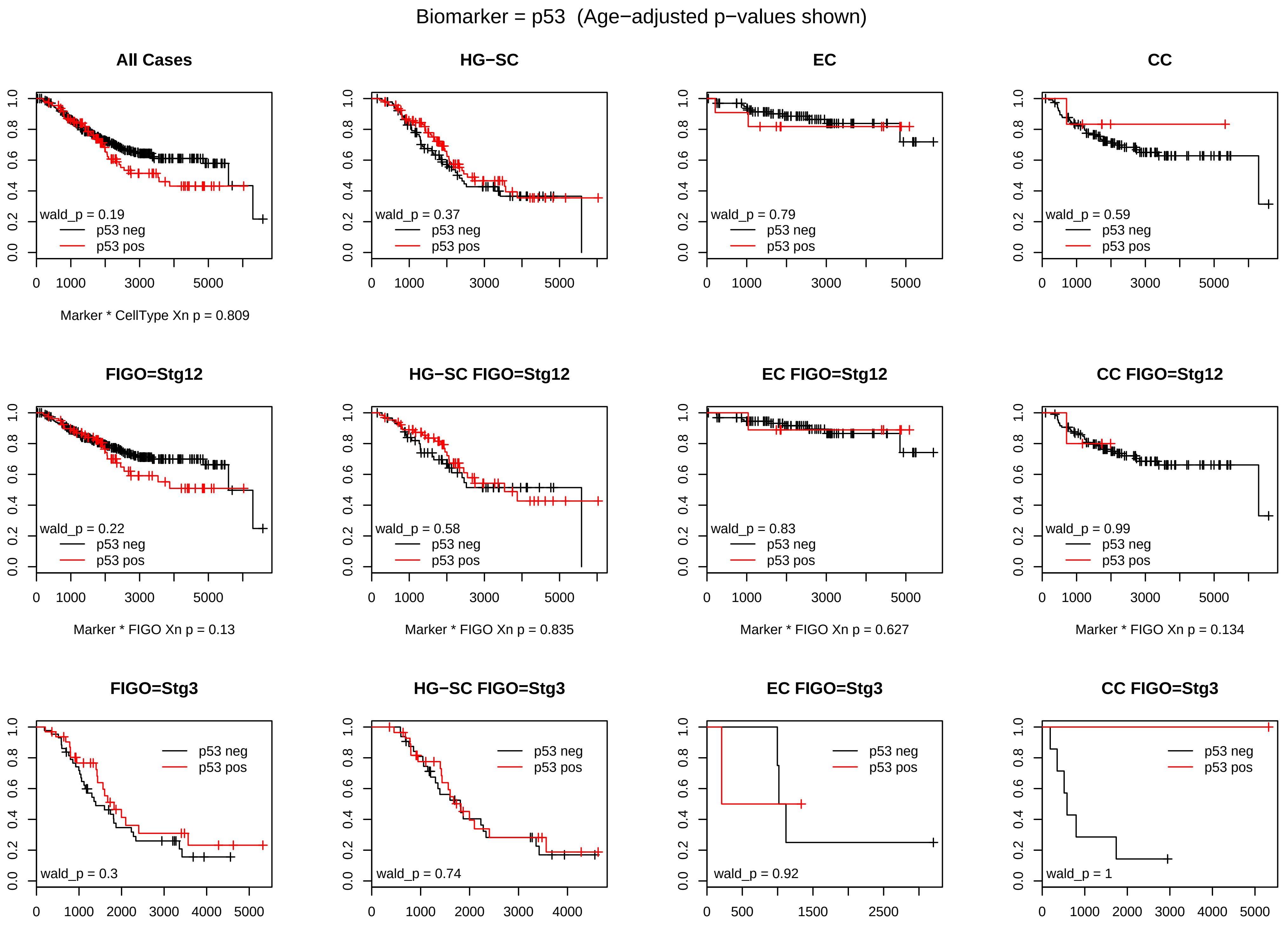
Biomarker = p53 (Age−adjusted p−values shown)
All Cases
wald_p = 0.19
p53 neg
p53 pos
Marker * CellType Xn p = 0.809
HG−SC
wald_p = 0.37
p53 neg
p53 pos
EC
wald_p = 0.79
p53 neg
p53 pos
CC
wald_p = 0.59
p53 neg
p53 pos
FIGO=Stg12
wald_p = 0.22
p53 neg
p53 pos
Marker * FIGO Xn p = 0.13
HG−SC FIGO=Stg12
wald_p = 0.58
p53 neg
p53 pos
Marker * FIGO Xn p = 0.835
EC FIGO=Stg12
wald_p = 0.83
p53 neg
p53 pos
Marker * FIGO Xn p = 0.627
CC FIGO=Stg12
wald_p = 0.99
p53 neg
p53 pos
Marker * FIGO Xn p = 0.134
FIGO=Stg3
p53 neg
p53 pos
wald_p = 0.3
HG−SC FIGO=Stg3
p53 neg
p53 pos
wald_p = 0.74
EC FIGO=Stg3
p53 neg
p53 pos
wald_p = 0.92
CC FIGO=Stg3
p53 neg
p53 pos
wald_p = 1

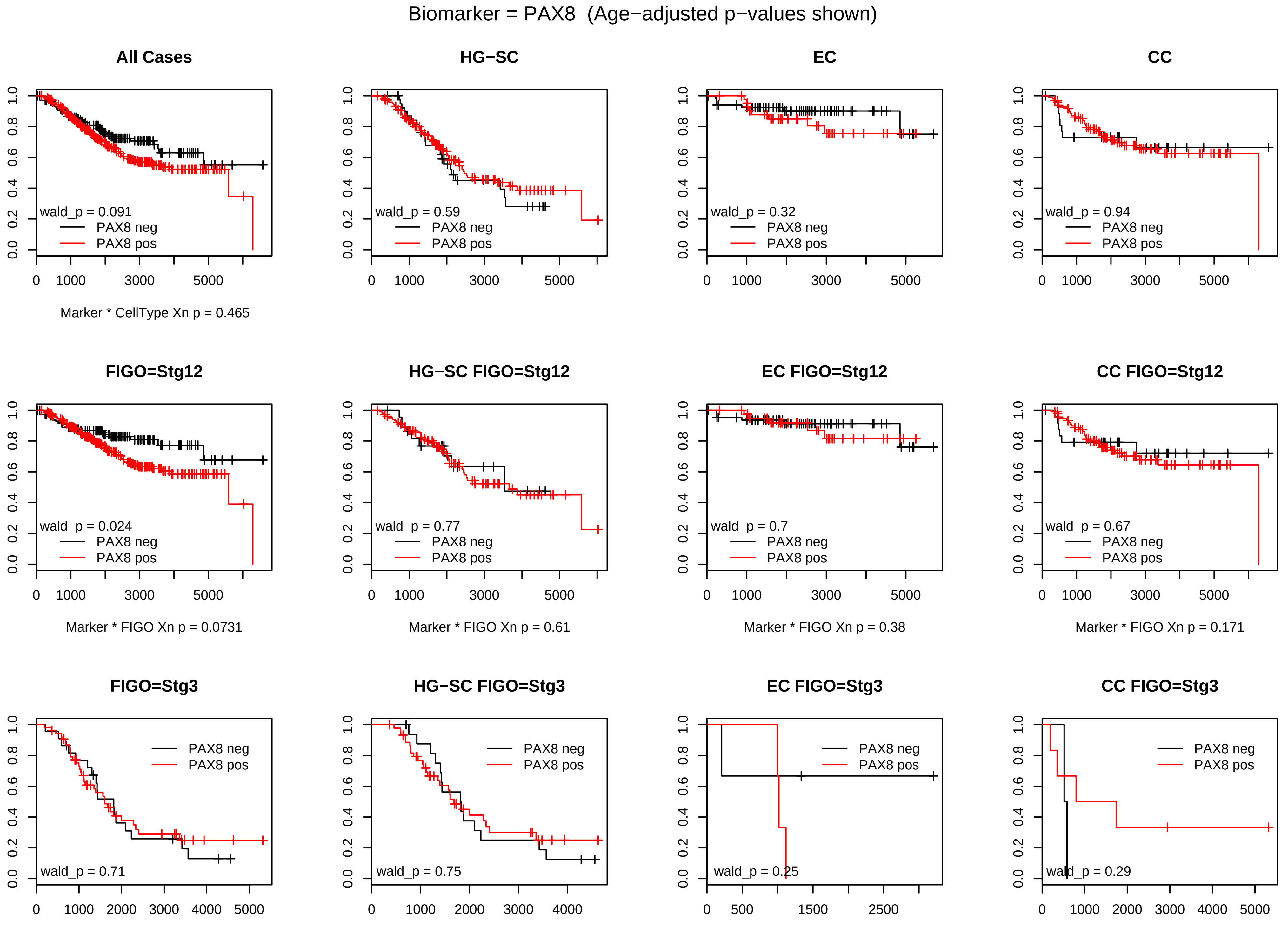
Biomarker = PAX8 (Age−adjusted p−values shown)
All Cases
wald_p = 0.091
PAX8 neg
PAX8 pos
Marker * CellType Xn p = 0.465
HG−SC
wald_p = 0.59
PAX8 neg
PAX8 pos
EC
wald_p = 0.32
PAX8 neg
PAX8 pos
CC
wald_p = 0.94
PAX8 neg
PAX8 pos
FIGO=Stg12
wald_p = 0.024
PAX8 neg
PAX8 pos
Marker * FIGO Xn p = 0.0731
HG−SC FIGO=Stg12
wald_p = 0.77
PAX8 neg
PAX8 pos
Marker * FIGO Xn p = 0.61
EC FIGO=Stg12
wald_p = 0.7
PAX8 neg
PAX8 pos
Marker * FIGO Xn p = 0.38
CC FIGO=Stg12
wald_p = 0.67
PAX8 neg
PAX8 pos
Marker * FIGO Xn p = 0.171
FIGO=Stg3
PAX8 neg
PAX8 pos
wald_p = 0.71
HG−SC FIGO=Stg3
PAX8 neg
PAX8 pos
wald_p = 0.75
EC FIGO=Stg3
PAX8 neg
PAX8 pos
wald_p = 0.25
CC FIGO=Stg3
PAX8 neg
PAX8 pos
wald_p = 0.29

Biomarker = PR (Age−adjusted p−values shown)

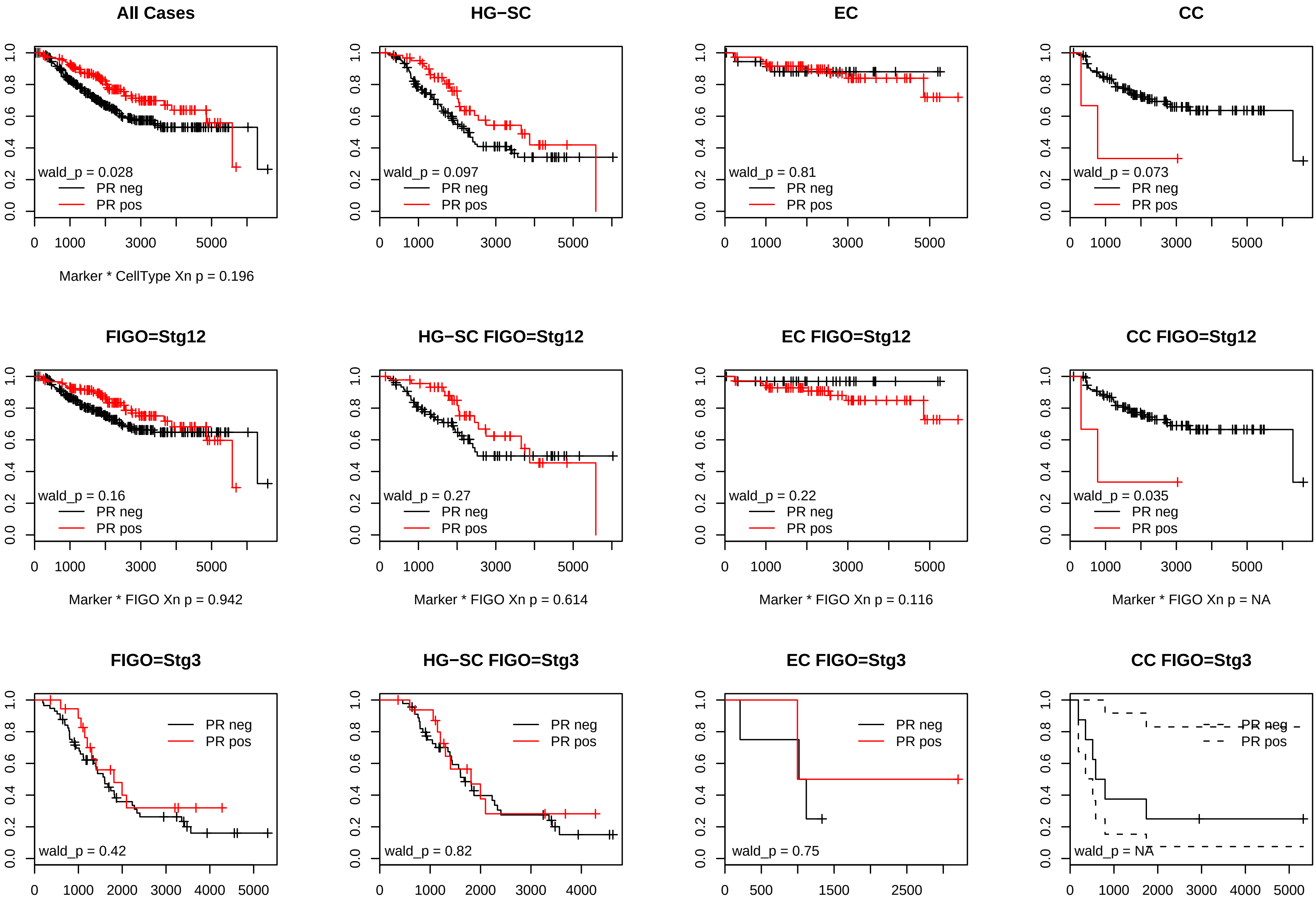

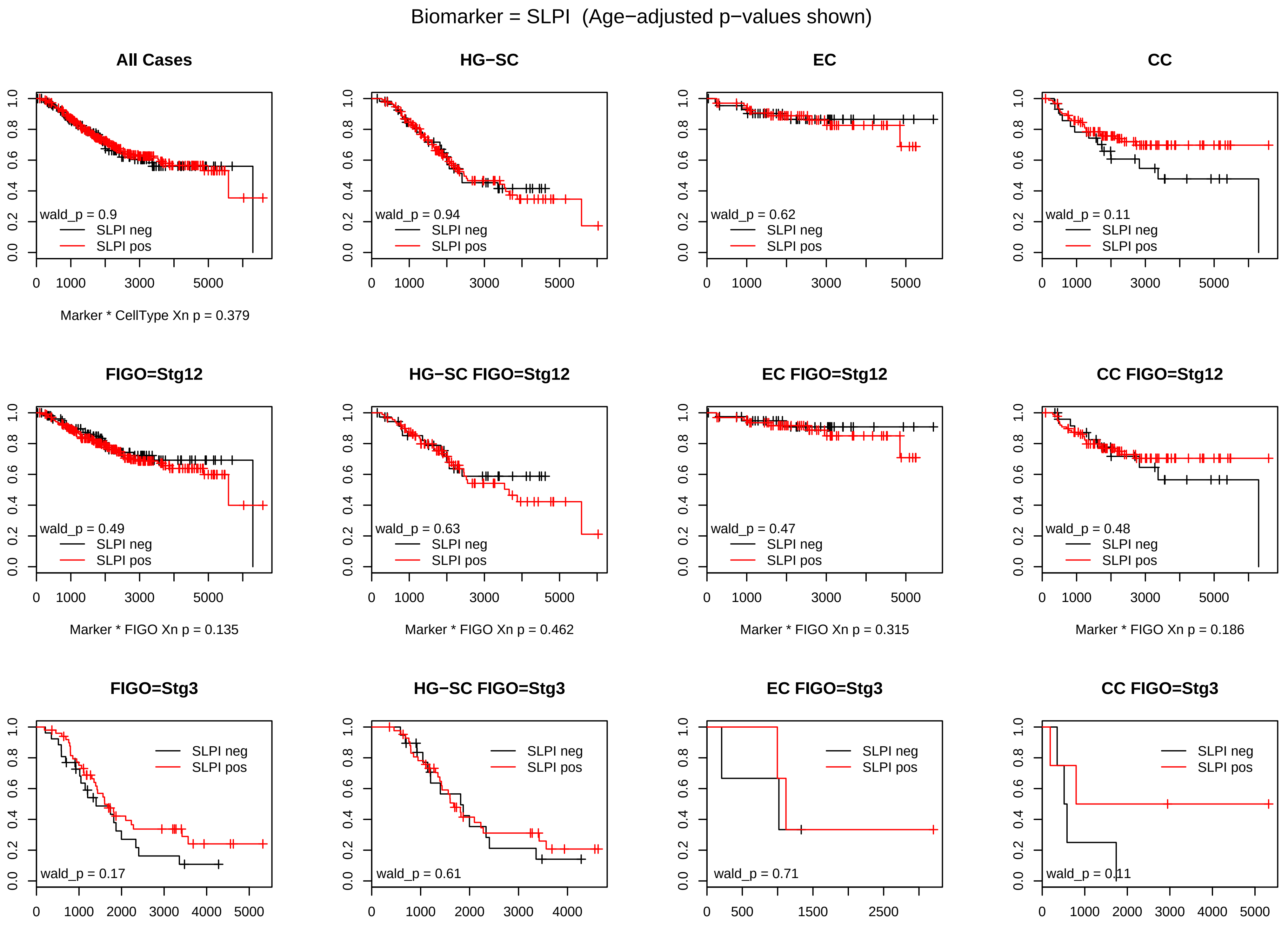

Biomarker = SLPI (Age−adjusted p−values shown)
All Cases
wald_p = 0.9
SLPI neg
SLPI pos
Marker * CellType Xn p = 0.379
HG−SC
wald_p = 0.94
SLPI neg
SLPI pos
EC
wald_p = 0.62
SLPI neg
SLPI pos
CC
wald_p = 0.11
SLPI neg
SLPI pos
FIGO=Stg12
wald_p = 0.49
SLPI neg
SLPI pos
Marker * FIGO Xn p = 0.135
HG−SC FIGO=Stg12
wald_p = 0.63
SLPI neg
SLPI pos
Marker * FIGO Xn p = 0.462
EC FIGO=Stg12
wald_p = 0.47
SLPI neg
SLPI pos
Marker * FIGO Xn p = 0.315
CC FIGO=Stg12
wald_p = 0.48
SLPI neg
SLPI pos
Marker * FIGO Xn p = 0.186
FIGO=Stg3
SLPI neg
SLPI pos
wald_p = 0.17
HG−SC FIGO=Stg3
SLPI neg
SLPI pos
wald_p = 0.61
EC FIGO=Stg3
SLPI neg
SLPI pos
wald_p = 0.71
CC FIGO=Stg3
SLPI neg
SLPI pos
wald_p = 0.11

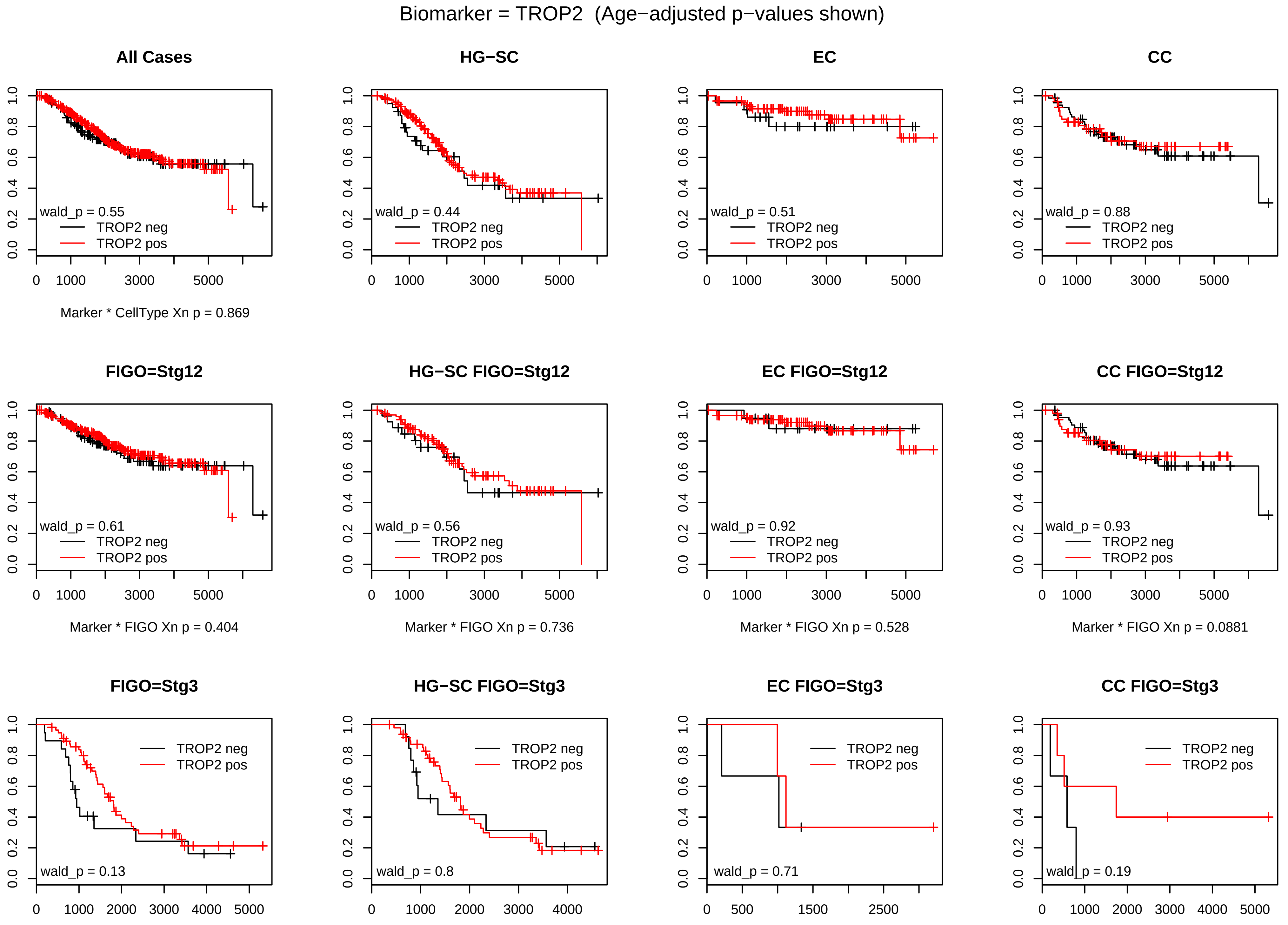
Biomarker = TROP2 (Age−adjusted p−values shown)
All Cases
wald_p = 0.55
TROP2 neg
TROP2 pos
Marker * CellType Xn p = 0.869
HG−SC
wald_p = 0.44
EC
wald_p = 0.51
CC
wald_p = 0.88
FIGO=Stg12
wald_p = 0.61
Marker * FIGO Xn p = 0.404
HG−SC FIGO=Stg12
wald_p = 0.56
Marker * FIGO Xn p = 0.736
EC FIGO=Stg12
wald_p = 0.92
Marker * FIGO Xn p = 0.528
CC FIGO=Stg12
wald_p = 0.93
Marker * FIGO Xn p = 0.0881
FIGO=Stg3
wald_p = 0.13
HG−SC FIGO=Stg3
wald_p = 0.8
EC FIGO=Stg3
wald_p = 0.71
CC FIGO=Stg3
wald_p = 0.19

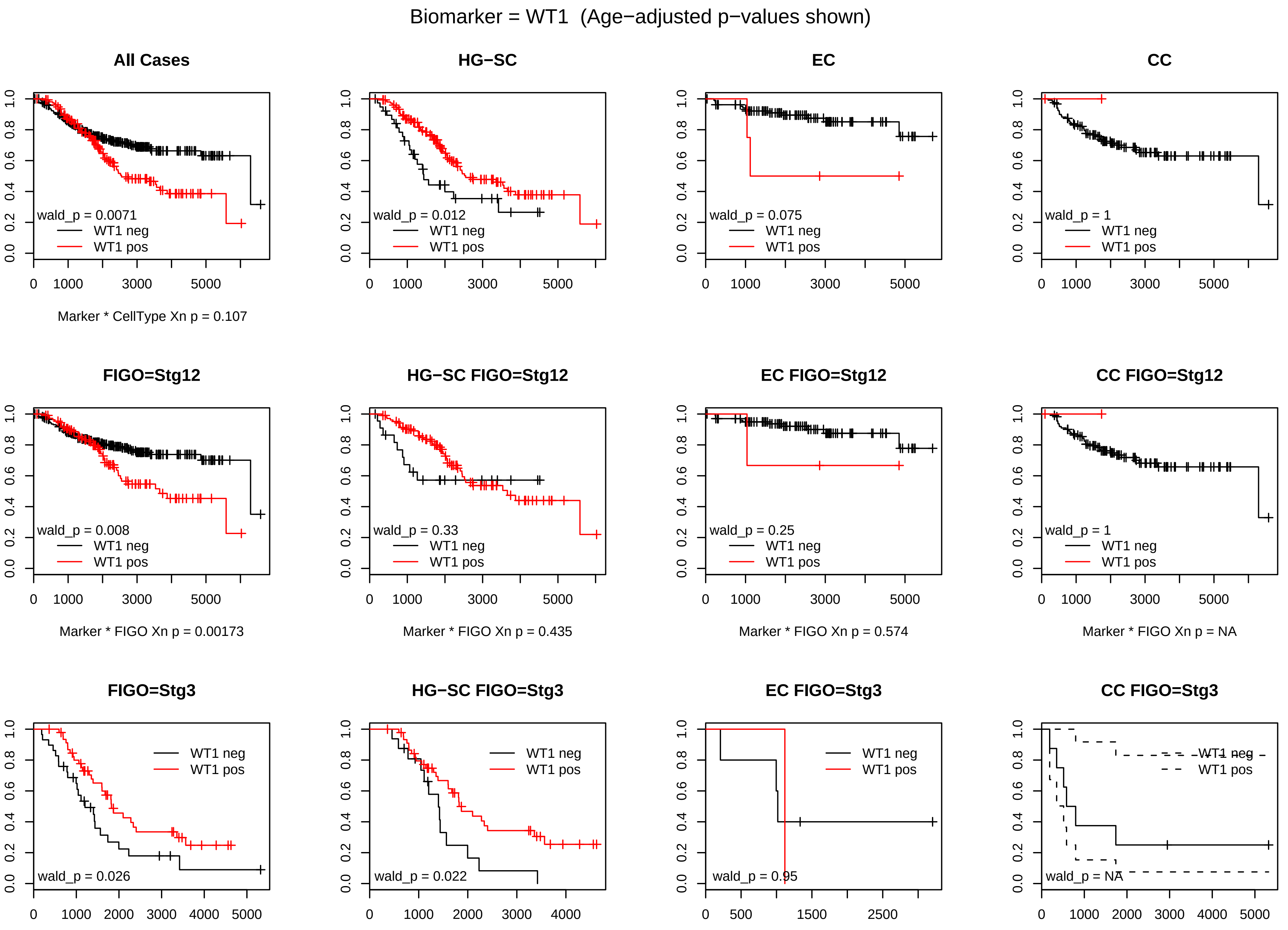
Biomarker = WT1 (Age−adjusted p−values shown)
All Cases
wald_p = 0.0071
WT1 neg
WT1 pos
Marker * CellType Xn p = 0.107
HG−SC
wald_p = 0.012
WT1 neg
WT1 pos
EC
wald_p = 0.075
WT1 neg
WT1 pos
CC
wald_p = 1
WT1 neg
WT1 pos
FIGO=Stg12
wald_p = 0.008
WT1 neg
WT1 pos
Marker * FIGO Xn p = 0.00173
HG−SC FIGO=Stg12
wald_p = 0.33
WT1 neg
WT1 pos
Marker * FIGO Xn p = 0.435
EC FIGO=Stg12
wald_p = 0.25
WT1 neg
WT1 pos
Marker * FIGO Xn p = 0.574
CC FIGO=Stg12
wald_p = 1
WT1 neg
WT1 pos
Marker * FIGO Xn p = NA
FIGO=Stg3
WT1 neg
WT1 pos
wald_p = 0.026
HG−SC FIGO=Stg3
WT1 neg
WT1 pos
wald_p = 0.022
EC FIGO=Stg3
WT1 neg
WT1 pos
wald_p = 0.95
CC FIGO=Stg3
WT1 neg
WT1 pos
wald_p = NA
