# Supplementary material for: Ovarian Carcinoma Subtypes Are Different Diseases: Implications for Biomarker Studies
Source: PLoS Med. 2008 Dec 2;5(12):e232. doi: 10.1371/journal.pmed.0050232 (PMC2592352; doi:10.1371/journal.pmed.0050232)
Supplement: Supplementary file 2 [file pmed.0050232.st001.doc]

Table S1 Definition of positive staining

| No. | Biomarker | Localisation | Cut-off for defining a case as positive |
| --- | --- | --- | --- |
| 1 | CA125 | membranous | ≥ 5% any intensity |
| 2 | CRABP-II | cytoplasmic | ≥ 5% moderate intensity |
| 3 | EpCam | membranous | ≥ 1% moderate intensity |
| 4 | ER | nuclear | ≥ 1% any intensity |
| 5 | F-Spondin | cytoplasmic | ≥ 1% moderate intensity |
| 6 | HE4 | membranous | ≥ 1% moderate intensity |
| 7 | IGF2 | cytoplasmic | ≥ 1% moderate intensity |
| 8 | K-Cadherin | membranous | ≥ 5% any intensity |
| 9 | Ki-67 | nuclear | median |
| 10 | KISS1 | cytoplasmic | ≥ 5% strong intensity |
| 11 | Matriptase | membranous | ≥ 1% moderate intensity |
| 12 | Mesothelin | membranous | ≥ 5% any intensity |
| 13 | MIF | cytoplasmic | ≥ 5% any intensity |
| 14 | MMP7 | cytoplasmic | ≥ 1% moderate intensity |
| 15 | p21 | nuclear | ≥ 1% any intensity |
| 16 | p53 | nuclear | ≥ 80% any intensity |
| 17 | PAX8 | nuclear | ≥ 1% any intensity |
| 18 | PR | nuclear | ≥ 1% any intensity |
| 19 | SLPI | cytoplasmic | ≥ 5% any intensity |
| 20 | TROP2 | membranous | ≥ 5% any intensity |
| 21 | WT1 | nuclear | ≥ 5% any intensity |
