# Supplementary material for: Ovarian Carcinoma Subtypes Are Different Diseases: Implications for Biomarker Studies
Source: PLoS Med. 2008 Dec 2;5(12):e232. doi: 10.1371/journal.pmed.0050232 (PMC2592352; doi:10.1371/journal.pmed.0050232)
Supplement: Supplementary file 3 [file pmed.0050232.st002.doc]

Table S2 Biomarker expression rate across stage, subtype and stage within subtypes.

|  | **Subtype** | All | | | | | **Pearson Chi Square test across stage** | | **High-grade serous** | | | | **Low-grade serous** | **Clear cell** | | | | **Endometrioid** | | | | **Mucinous** | | | **Pearson Chi Square test across Subtype**  **(All stages)** | |
| --- | --- | --- | --- | --- | --- | --- | --- | --- | --- | --- | --- | --- | --- | --- | --- | --- | --- | --- | --- | --- | --- | --- | --- | --- | --- | --- |
|  | Stage | **All** | | **I** | **II** | **III** |  |  | All | I | II | III | All | All | I | II | III | All | I | II | III | All | I | II/III |  |  |
|  | ***N*** | ***500*** | ***500*** | ***205*** | ***211*** | ***84*** |  |  | *200* | *49* | *85* | *66* | *12* | *132* | *68* | *56* | *8* | *125* | *69* | *50* | *6* | *31* | *18* | *13* |  |  |
|  |  | ***N*** | % | % | % | % | *P-value** | *P-value*** | % | % | % | % | % | % | % | % | % | % | % | % | % | % | % | % | *P-value** | *P-value *** |
| ***1*** | ***CA125*** | *479* | 76.6 | 74.4 | 76.4 | 81.7 | 0.47 | 0.90 | 88.2 | 95.8 | 87.7 | 83.1 | 100.0 | 67.2 | 68.8 | 64.2 | 75.0 | 81.1 | 81.2 | 83.0 | 66.7 | 7.4 | 6.3 | 9.1 | <0.0001 | 0.0002 |
| ***2*** | ***CRABP-II*** | *488* | 33.8 | 29.4 | 30.5 | 52.4 | 0.00055 | 0.0016 | 49.7 | 43.8 | 45.2 | 60.0 | 27.3 | 30.7 | 35.4 | 25.9 | 25.0 | 20.2 | 21.7 | 14.3 | 50.0 | 0.0 | 0.0 | 0.0 | <0.0001 | 0.0002 |
| ***3*** | ***EpCam*** | *440* | 82.7 | 86.2 | 78.5 | 84.9 | 0.13 | 0.26 | 79.4 | 81.1 | 74.4 | 74.2 | 72.7 | 89.2 | 91.4 | 85.1 | 100.0 | 81.7 | 82.5 | 80.9 | 80.0 | 85.2 | 93.8 | 72.7 | 0.23 | .46 |
| ***4*** | ***ER*** | *489* | 54.6 | 47.8 | 56.2 | 67.5 | 0.0084 | 0.0202 | 75.8 | 73.5 | 77.4 | 75.4 | 80.0 | 10.2 | 12.1 | 9.3 | 0.0 | 77.0 | 75.4 | 83.0 | 50.0 | 3.3 | 0.0 | 8.3 | <0.0001 | 0.0002 |
| ***5*** | ***F-Spondin*** | *362* | 38.1 | 30.1 | 35.9 | 60.6 | 0.00013 | 0.0002 | 57.9 | 57.6 | 52.2 | 64.7 | 50.0 | 33.0 | 34.8 | 27.8 | 50.0 | 16.5 | 10.0 | 21.0 | 40.0 | 17.4 | 15.4 | 20.0 | <0.0001 | 0.0002 |
| ***6*** | ***HE4*** | *369* | 49.9 | 46.6 | 48.7 | 59.7 | 0.19 | 0.39 | 62.4 | 67.6 | 57.1 | 65.4 | 37.5 | 17.8 | 19.1 | 18.9 | 0.0 | 68.7 | 66.0 | 70.7 | 80.0 | 13.6 | 7.7 | 22.2 | <0.0001 | 0.0002 |
| ***7*** | ***IGF2*** | *488* | 60.0 | 61.9 | 67.7 | 73.8 | 0.14 | 0.28 | 76.1 | 79.6 | 75.0 | 75.0 | 60.0 | 44.6 | 48.5 | 38.9 | 50.0 | 54.5 | 52.2 | 58.7 | 50.0 | 41.4 | 38.9 | 45.5 | 0.00069 | 0.0012 |
| ***8*** | ***K_Cadherin*** | *485* | 42.7 | 33.3 | 41.4 | 67.9 | <0.0001 | 0.0002 | 69.9 | 72.3 | 65.6 | 73.8 | 45.5 | 26.8 | 23.4 | 25.5 | 62.5 | 24.6 | 24.6 | 23.4 | 33.3 | 3.6 | 0.0 | 9.1 | <0.0001 | 0.0002 |
| ***9*** | ***Ki-67 Median 95% CI*** | *451* | 13.0  1.0-60.3 | 10.1  1.0-55.7 | 13.0  1.0-65.7 | 20.5  0.8-63.5 | 0.0010 | 0.0028 | 22.4  3.6-69.9 | 22.6  5.9-70.6 | 21.5  4.0-74.7 | 22.2  2.5-66.2 | 2.5  0.5-20.4 | 7.6  0.5-45.0 | 7.5  0.8-27.1 | 7.8  0.4-64.7 | 11.1  0-49.9 | 8.2  0.8-49.0 | 8.2  0.8-48.7 | 7.2  0.8-47.9 | 20.5  0.8-56.0 | 12.9  2.1-60.9 | 13.7  1.9-72.7 | 9.3  2.3-48.1 | <0.0001 | 0.0002 |
| ***10*** | ***KISS1*** | *478* | 9.2 | 12.3 | 8.5 | 3.6 | 0.063 | 0.12 | 1.6 | 0.0 | 3.7 | 0.0 | 0.0 | 21.9 | 22.7 | 20.4 | 25.0 | 8.1 | 11.8 | 4.1 | 0.0 | 8.3 | 6.7 | 11.1 | <0.0001 | 0.0002 |
| ***11*** | ***Matriptase*** | *362* | 49.7 | 58.6 | 42.7 | 46.3 | 0.019 | 0.037 | 45.6 | 59.4 | 38.1 | 46.2 | 37.5 | 28.7 | 37.0 | 17.1 | 33.0 | 67.0 | 69.8 | 64.1 | 60.0 | 86.4 | 92.3 | 77.8 | <0.0001 | 0.0002 |
| ***12*** | ***Mesothelin*** | *493* | 49.2 | 36.1 | 55.6 | 65.5 | <0.0001 | 0.0002 | 79.9 | 79.6 | 84.7 | 73.8 | 81.8 | 32.8 | 27.9 | 40.0 | 25.0 | 26.6 | 21.7 | 34.7 | 16.7 | 0.0 | 0.0 | 0.0 | <0.0001 | 0.0002 |
| ***13*** | ***MIF*** | *485* | 65.3 | 58.3 | 54.1 | 56.6 | 0.70 | 0.60 | 60.5 | 73.9 | 56.0 | 56.9 | 63.6 | 54.7 | 55.4 | 54.5 | 50.0 | 57.7 | 58.0 | 57.1 | 60.0 | 24.1 | 27.8 | 18.2 | 0.013 | 0.023 |
| ***14*** | ***MMP7*** | *487* | 38.6 | 44.5 | 35.6 | 31.7 | 0.071 | 0.14 | 30.2 | 38.3 | 23.5 | 33.3 | 27.3 | 25.8 | 21.5 | 32.7 | 12.5 | 63.4 | 71.0 | 56.3 | 33.3 | 48.3 | 44.4 | 54.5 | <0.0001 | 0.0002 |
| ***15*** | ***p21*** | *493* | 51.7 | 59.3 | 50.7 | 35.7 | 0.0012 | 0.0022 | 33.5 | 37.5 | 32.1 | 32.1 | 54.5 | 73.5 | 79.4 | 67.9 | 62.5 | 62.1 | 63.8 | 61.2 | 50.0 | 31.0 | 27.8 | 40.0 | <0.0001 | 0.0002 |
| ***16*** | ***p53*** | *485* | 25.4 | 15.8 | 28.5 | 41.5 | <0.0001 | 0.0002 | 50.5 | 40.8 | 59.3 | 46.9 | 20.0 | 6.2 | 4.5 | 7.4 | 12.5 | 9.8 | 8.7 | 8.3 | 33.3 | 7.1 | 11.8 | 0.0 | <0.0001 | 0.0002 |
| ***17*** | ***PAX8*** | *493* | 63.0 | 34.8 | 40.8 | 49.4 | 0.062 | 0.113 | 79.7 | 81.3 | 83.3 | 73.8 | 70.0 | 76.5 | 75.0 | 78.6 | 75.0 | 38.7 | 39.1 | 36.7 | 50.0 | 6.9 | 0.0 | 18.2 | <0.0001 | 0.0002 |
| ***18*** | ***PR*** | *486* | 33.7 | 33.3 | 35.8 | 29.87 | 0.62 | 0.77 | 34.7 | 39.1 | 37.8 | 27.7 | 70.0 | 2.3 | 1.5 | 3.7 | 0.0 | 66.7 | 66.7 | 70.8 | 30.3 | 13.3 | 5.6 | 25.0 | <0.0001 | 0.0002 |
| ***19*** | ***SLPI*** | *490* | 65.1 | 65.3 | 64.2 | 66.7 | 0.93 | 0.15 | 71.1 | 75.0 | 69.8 | 70.8 | 63.6 | 74.6 | 77.6 | 74.5 | 50.0 | 59.3 | 60.9 | 58.3 | 50.0 | 3.6 | 5.9 | 0.0 | <0.0001 | 0.0002 |
| ***20*** | ***TROP2*** | *491* | 67.0 | 68.0 | 62.7 | 75.0 | 0.12 | 0.26 | 77.7 | 79.6 | 77.1 | 76.9 | 90.9 | 43.4 | 48.5 | 34.5 | 62.5 | 77.4 | 81.2 | 75.5 | 50.0 | 44.8 | 55.6 | 27.3 | <0.0001 | 0.0002 |
| ***21*** | ***WT1*** | *493* | 35.3 | 20.5 | 39.0 | 62.7 | <0.0001 | 0.0002 | 79.9 | 77.6 | 84.7 | 75.4 | 70.0 | 2.3 | 2.9 | 1.9 | 0.0 | 4.1 | 1.4 | 6.3 | 16.7 | 0.0 | 0.0 | 0.0 | <0.0001 | 0.0002 |

Values are given as percentage of positive cases, Ki-67 as continuous variable is shown as median with 95% confidence interval (CI).

P-value* – raw unadjusted asymptotic p-values by Fisher exact test (Kruskal-Wallis test for Ki-67), P-value** permutation test p-value, two-sided p-value are shown.
