# Supplementary material for: Ovarian Carcinoma Subtypes Are Different Diseases: Implications for Biomarker Studies
Source: PLoS Med. 2008 Dec 2;5(12):e232. doi: 10.1371/journal.pmed.0050232 (PMC2592352; doi:10.1371/journal.pmed.0050232)
Supplement: Supplementary file 4 [file pmed.0050232.st003.doc]

Table S3 Multivariable COX proportional hazards including stage, subtype and age for the entire cohort.

| **Variable** | **Risk factor** | **RR (95% CI)** | **P-Value** |
| --- | --- | --- | --- |
| Age |  |  | 0.211 |
| Stage | 1 | 1.0 | <0.0001 |
|  | 2 | 1.30 (0.86-1.93) |  |
|  | 3 | 3.00 (1.87-4.66) |  |
| Subtype | Endometrioid | 1.0 | 0.015 |
|  | Mucinous | 2.13 (0.87-5.18) |  |
|  | Low-grade serous | 2.30 (0.76-6.99) |  |
|  | Clear Cell | 2.31 (1.29-4.15) |  |
|  | High-grade serous | 2.74 (1.56-4.81) |  |

RR – risk ratio. CI – confidence interval

No RR is calculated for age because it is used as continuous variable.
