# Supplementary material for: Ovarian Carcinoma Subtypes Are Different Diseases: Implications for Biomarker Studies
Source: PLoS Med. 2008 Dec 2;5(12):e232. doi: 10.1371/journal.pmed.0050232 (PMC2592352; doi:10.1371/journal.pmed.0050232)
Supplement: Supplementary file 5 [file pmed.0050232.st004.doc]

Table S4 Multivariable COX proportional hazards including stage, subtype, age and WT-1 for the entire cohort and high-grade serous carcinomas.

| **Variable** | **Risk factor** | **Entire cohort** | | **High-grade serous** | |
| --- | --- | --- | --- | --- | --- |
|  |  | **RR (95% CI)** | **P-Value** | **RR (95% CI)** | **P-Value** |
| Age |  |  | 0.225 |  | 0.190 |
| Stage | 1 | 1.0 | <0.0001 | 1.0 | 0.002 |
|  | 2 | 1.30 (0.86-1.94) |  | 0.86 (0.48-1.54) |  |
|  | 3 | 3.00 (1.87-4.70) |  | 1.96 (1.12-3.40) |  |
| Subtype | Endometrioid | 1.0 | 0.010 |  |  |
|  | Mucinous | 2.01 (0.85-5.02) |  |  |  |
|  | Low-grade serous | 2.18 (0.62-7.72) |  |  |  |
|  | Clear Cell | 2.30 (1.28-4.12) |  |  |  |
|  | High-grade serous | 3.40 (1.73-6.69) |  |  |  |
| WT1 | Negative | 1.0 | 0.20 | 1.0 | 0.017 |
|  | Positive | 0.73 (0.45-1.18) |  | 0.55 (0.34-0.90) |  |

RR – risk ratio. CI – confidence interval

No RR is calculated for age because it is used as continuous variable.
